# Advances in the Robustness of Wearable Electronic Textiles: Strategies, Stability, Washability and Perspective

**DOI:** 10.3390/nano12122039

**Published:** 2022-06-14

**Authors:** Mohammad Shak Sadi, Eglė Kumpikaitė

**Affiliations:** Department of Production Engineering, Faculty of Mechanical Engineering and Design, Kaunas University of Technology, Studentų Str. 56, LT-51424 Kaunas, Lithuania; mohammad.shak@ktu.edu

**Keywords:** e-textiles, strategies, stability, washability, assessment protocols, standardization

## Abstract

Flexible electronic textiles are the future of wearable technology with a diverse application potential inspired by the Internet of Things (IoT) to improve all aspects of wearer life by replacing traditional bulky, rigid, and uncomfortable wearable electronics. The inherently prominent characteristics exhibited by textile substrates make them ideal candidates for designing user-friendly wearable electronic textiles for high-end variant applications. Textile substrates (fiber, yarn, fabric, and garment) combined with nanostructured electroactive materials provide a universal pathway for the researcher to construct advanced wearable electronics compatible with the human body and other circumstances. However, e-textiles are found to be vulnerable to physical deformation induced during repeated wash and wear. Thus, e-textiles need to be robust enough to withstand such challenges involved in designing a reliable product and require more attention for substantial advancement in stability and washability. As a step toward reliable devices, we present this comprehensive review of the state-of-the-art advances in substrate geometries, modification, fabrication, and standardized washing strategies to predict a roadmap toward sustainability. Furthermore, current challenges, opportunities, and future aspects of durable e-textiles development are envisioned to provide a conclusive pathway for researchers to conduct advanced studies.

## 1. Introduction

**Figure 1:**
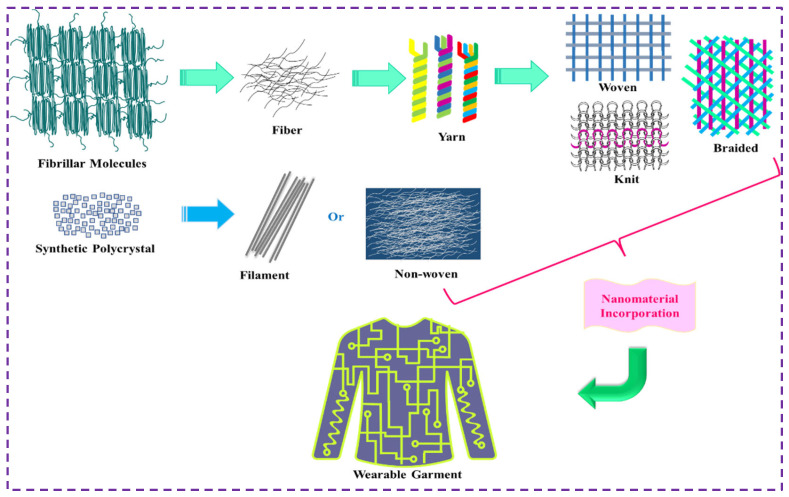
Hierarchy of textile structures.

Wearable technologies have created a universal platform for innovation and advancement towards the 4th industrial revolution in versatile areas to connect the virtual world with reality. Wearable electronics can facilitate human quality of life in all possible aspects by properly monitoring different actions in real time [1]. However, the traditional electronic components are often rigid, uncomfortable, and difficult to integrate with the complicated architecture of the human body, which substantially limits their practical application [2,3]. Textile materials (clothing) are always worn by the wearer and are considered the most ideal platform for designing and incorporating electronics without compromising comfort and aesthetics. The smart textiles are capable of sensing, reacting, and adapting to external events or stimuli to capture, process, and analyze data remotely using electronics built with e-textiles and can be used for wearable applications [4]. E-textiles constructed of fibrous textile materials are expected to exhibit their inherent characteristics (i.e., comfortability, flexibility, stretchability, breathability, light weight, etc.) when there is no alteration of properties involved in the fabrication process. Moreover, e-textiles can be adapted to any sophisticated electronic components [5]. Besides, e-textiles can be constructed with various hierarchical architectures (Figure 1) in the form of fiber, yarn, fabric, and garments to facilitate the application perspective in future high-end miniature electronics. Levi’s in collaboration with Philips introduced the first commercial wearable e-textile (jacket) in summer 2000 [6]. Since then, e-textiles are of great interest and have experienced disruptive innovation and advancement in terms of research and application. So far, e-textiles have been proposed to be utilized in different areas, i.e., healthcare [7,8], sensing [9,10], defense [11,12], sports [13,14], personal protection [15,16], fashion [17,18], energy [19,20], thermal management [21,22], magnetic shielding [23,24], communication [25,26], etc. The incorporation of metal nanoparticles (silver [27,28], gold [29,30], copper [31,32], zinc oxide [33,34], gallium [35,36], platinum [37,38], aluminum [39,40], nickel [41,42], cobalt [43,44], tin [45,46], etc.), carbon nanomaterials (carbon nanotube [47,48], graphene [49,50], carbon black [51,52], activated carbon [53,54], etc.), conductive polymers (Polypyrrole-PPy [55,56], Polyaniline-PANI [57,58], poly(3,4-ethylenedioxythiophene) polystyrene sulfonate-PEDOT: PSS [59,60], etc.) and other 2D materials (MXene [61,62], TMD [63,64], etc.) with textile substrates (non-conductive in nature) is an important aspect of e-textiles fabrication. The electrically functionalized textile substrate of different forms ranging from fiber/filament to fabric/garment can be achieved via different approaches, i.e., coating (dip-coating [65,66], spray coating [67,68], ultrasonic coating [69,70], knife coating [71,72], spin coating [73,74], etc.), printing (screen printing [75,76], inkjet printing [77,78], extrusion printing [79,80], gravure printing [81,82], laser printing [83,84], stencil printing [85,86], 3D printing [87,88], etc.), electrospinning (melt spinning [89,90], dry spinning [91,92], wet spinning [93,94], etc.), electrodeposition [95,96], polymerization [97], thin-film deposition [98,99], nanopattern [100], etc. The conductive materials adhere to intrinsically nonpolar textile materials mainly through physical absorption [101] and mostly fail (detach or decay from the substrate surface) to comply with different actions of the wearer, i.e., bending, twisting, friction, etc. Different approaches with advanced material processing and chemistry are availed to alleviate such challenges in designing practically viable and wearable devices.

To date, many high-performance e-textiles with improved performance are reported [102,103,104,105,106], however, poor stability and washability have been the major challenges restricting their true practical essence. Moreover, the incompetent durability of the e-textiles may encounter environmental and safety concerns by releasing toxic organic/inorganic nanostructured compounds to the ecosystem and wearer [107]. Hence, to improve stability and washability, variant structures have been proposed that differ from material choice, modification, fabrication, and even assessment protocols. Although much research claimed that their constructed e-textile components had superior durability, they could barely withstand repeated laundry and mechanical deformation for a long time without compromising their electro-conductive properties [108,109]. It is evident that remarkable progress has been achieved toward sustainable e-textiles products, but there remains a large gap in the adopted tactics and output reliability. There are a limited number of standard wash assessment protocols entirely focused on e-textiles present in academia. So, individually designed wash assessment protocols along with traditional approaches are being followed to verify researcher interest in this regard. In many cases, the product is claimed to be durable despite exhibiting poor fastness, and some research even claimed durability without stability and wash tests [110,111,112]. Therefore, reliable and standardized wash protocols with defined circumstances and evaluation criteria will facilitate the researcher’s ability to predict their product behavior and make reliable comparability of different e-textiles.

So far, various review articles have been published [113,114,115,116,117,118,119] mostly focusing on materials, fabrication strategies, architecture, multifunctional properties, and the application perspective of the wearable e-textiles, ignoring the importance of wash durability enhancement. Very few review articles [120,121,122] are available in the academia that entirely focus on washability and attempt to summarize different wash strategies, influencing parameters, and enhancement opportunities, but lack a pragmatic review that favors the improvement of the reliability and washability of e-textiles. Thus, this review summarized the most advanced multidisciplinary approaches from the substrate to consumer product design with regard to advanced stability, washability, and explained different aspects of washing features, leading toward standardized evaluation protocols. Initially, recently developed durable e-textiles of different hierarchical structures (fiber, yarn, fabric/garment) are discussed along with the state-of-the-art advances in reliable device fabrication. Afterward, all aspects of the stability and wash durability are addressed, from traditional testing to the establishment of standardized protocols. In the end, the remaining challenges, opportunities, and the future perspective of this area are discussed. This comprehensive review is expected to tremendously facilitate a proper understanding of this area and open a new direction for the research community toward the evolution of durable electronic textile components.

## 2. Architecture of E-Textiles

**Figure 2:**
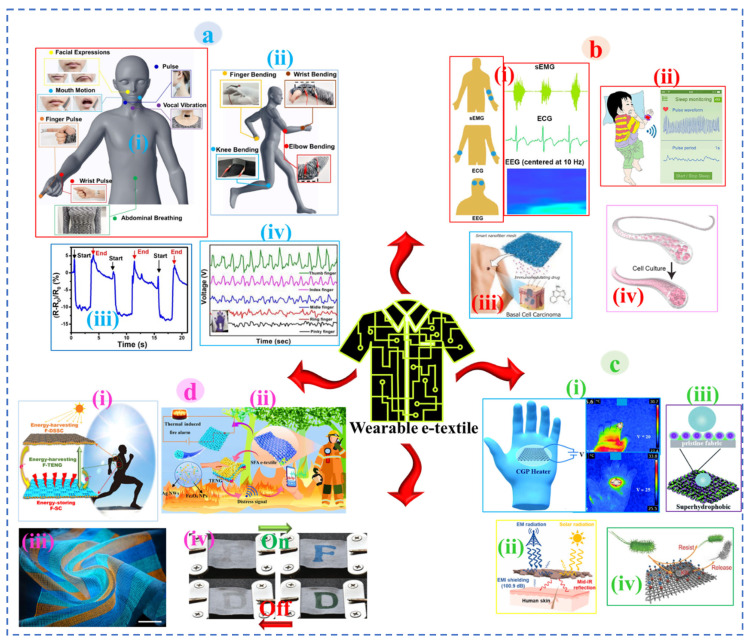
Different areas of application of e-textiles. (**a**) Human motion monitoring. (i) facial expression, vocal vibration, breathing, different pulse. (ii) different joints (finger, wrist, knee, elbow) motion, (iii) laughing. Reproduced with permission [133]. Copyright 2018, American Chemical Society. (iv) Signals originated from different finger bending. Reproduced with permission [134]. Copyright 2022, Elsevier. (**b**) Healthcare applications. (i) Monitoring of EMG, ECG, and EEG. Reproduced with permission [135]. Copyright 2020, American Chemical Society. (ii) Sleep monitoring. Reproduced with permission [136]. Copyright 2020, Elsevier. (iii) Drug delivery. Reproduced with permission [137]. Copyright 2020, Merck KGaA, Darmstadt, Germany. (iv) Cell culture. Reproduced with permission [138]. Copyright 2013, Nature. (**c**) (i) Thermal heating. Reproduced with permission [139]. Copyright 2020, Royal Society of Chemistry. (ii) EMI shielding. Reproduced with permission [140]. Copyright 2021, Elsevier. (iii) Antimicrobial application. Reproduced with permission [141]. Copyright 2016, Wiley. (iv) Self-cleaning. Reproduced with permission [142]. Copyright 2016, Royal Society of Chemistry. (**d**) (i) Energy harvesting. Reproduced with permission [143]. Copyright 2016, Science. (ii) Fire alarm. Reproduced with permission [144]. Copyright 2022, American Chemical Society. (iii) Electronic display. Reproduced with permission [145]. Copyright 2021, Nature. (iv) Color-changing e-textiles. Reproduced with permission [146]. Copyright 2016, Royal Society of Chemistry.

E-textiles are the traditional textiles of different hierarchies embedded with multifunctional nanomaterials to be utilized in different areas, for instance, human motion monitoring, i.e., joints bending, walking, running, facial expression, vocal vibration, pulse, breathing, laughing, etc. (Figure 2a), healthcare applications, i.e., EMG, ECG, EEG, sleep monitoring, drug delivery, cell culture, etc. (Figure 2b), thermal heating (Figure 2c-i), electromagnetic shielding (Figure 2c-ii), antimicrobial protection (Figure 2c-iii), self-cleaning (Figure 2c-iv), energy storage/harvesting (Figure 2d-i), fire alarm (Figure 2d-ii), electronic display (Figure 2d-iii), color-changing (Figure 2d-iv), etc. with a wide spectrum of functions by mitigating the wear complexities associated with non-flexible and bulky wearable electronics. In other words, e-textiles can be electronically integrated textiles built with different responsive electronic components to sense, react, and adapt themselves in a given circumstance [123]. In a wearable e-garment, different sensors [124,125] and actuators [126,127] that are necessarily made of textiles are embedded and connected to a flexible power supply (fibrous supercapacitor [128,129], solar cell [130], nanogenerator [131,132], etc.) data processor, along with an external communication platform (Wi-Fi) for the acquired data to be further processed and monitored remotely. All these components are interconnected with each other using conductive yarn/line and are woven into the garment for wearable application. However, the electronic components built in textiles attached to the garment or clothing must exhibit similar characteristics in terms of stretchability, flexibility, sensitivity, and comfort against the skin of the wearer. Traditional textile materials with conductive features imparted by nanomaterials are used as a terminal to employ electronics and design wearable devices. The inadequate durability of nanomaterials’ coating on textiles, causing poor stability of electroconductive properties against human body joint-induced mechanical deformation, chemical phenomena (sweat, blood, liquid, detergent), and other challenges that may be involved in daily life, restricts the commercial essence of e-textiles. Thus, designing washable and durable e-textiles is the key to satisfy consumer requirements for future wearable e-textile devices with consistent performance in daily life events with comfort. The washability of e-textiles depends on the geometry of adopted textile interfaces, that is, 1D (fiber, yarn, filament), 2D (warp/weft knitted, woven, nonwoven), and 3D (triaxial composite structure, braided). Thus, e-textiles with a reliable substrate and architecture are important and are substantially examined for washable component design, which is briefly outlined in the next section.

### 2.1. Fiber Shaped Durable E-Textiles

Fiber is the first phase of the textile hierarchy which serves as the basic construction block of e-textiles, conductivity at the fiber level facilitates seamless integration of electronic function for the next generation of miniature devices. Nanomaterials with fiber components are expected to exhibit strong adhesion at the molecular level with improved electrical properties, mechanical properties (strength, flexibility, stretchability), durability (stability, washability), comfort, etc. Fiber materials can be made of natural (cellulose, protein) or synthetic resources. Synthetic fibers (the filament, i.e., continuous fibrous strand or nanofiber) are manufactured from polymer solution following different electrospinning processes. Traditional cellulosic textile fiber can be functionalized in the typical yarn manufacturing phase (sliver/roving) and subsequently spun into yarn.

**Figure 3:**
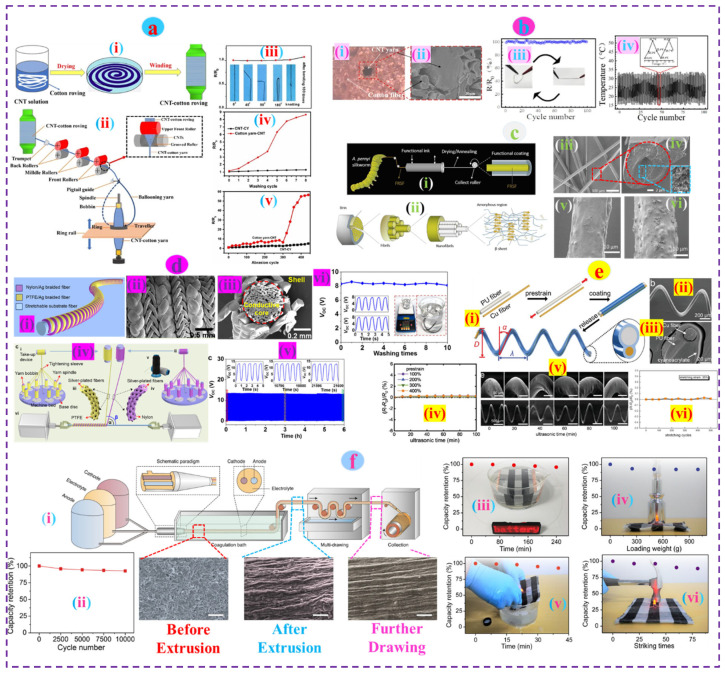
(**a**) Schematic of the roving coating technique (i) and (ii) ring spinning process, resistance change upon repeated bending (iii), washing (iv), and abrasion cycles (v). Reproduced with permission [147] Copyright 2018, Springer. (**b**) Optical image (i) and (ii) SEM image of the core-spun yarn, (iii) change of temperature as a function of time, and (iv) cyclic stability under 100 bending cycles. Reproduced with permission [148] Copyright 2022, Springer. (**c**) (i) Schematic of the functionalized silk fiber production, (ii) Structural hierarchy of the developed fiber, (iii,iv) SEM images of the fiber and cross-section, (v,vi) Surface morphology before and after 48 h of wash. Reproduced with permission [150] Copyright 2022, Elsevier. (**d**) (i) Schematic of the stretchable helical fiber, (ii) SEM images of the fiber surface, and (iii) cross-section, (iv) different steps involved in helical fiber manufacturing, (v) output voltage under repeated stretching-releasing, and (vi) washing. Reproduced with permission [152] Copyright 2022, ACS publications. (**e**) (i) Fabrication of 3D helical fiber, SEM images of the fiber (ii) and its cross-section (iii), electrical resistance upon ultrasonic washing (iv) and after wash surface morphology (v), cyclic stability under 35% strain (vi). Reproduced with permission [153] Copyright 2020, Wiley. (**f**) (i) Schematic of the fiber batteries production process with corresponding fiber morphologies at different phases of manufacturing, (ii) capacity retention under cyclic operations, and (iii,vi) durability underwater immersion, pressing, washing, and striking respectively. Reproduced with permission [154] Copyright 2022, Nature.

Yang et al. demonstrated that the incorporation of nanomaterials at the roving level gives the ring-spun yarn improved stability and washability compared to the cotton yarn coated with carbon nanotube (CNT) via the dip-coating technique. The roving modified ring-spun yarn can withstand repeated bending (180°) of 100 cycles with nominal resistance change (<10%), optimum stability for abrasion (up to 400 cycles), and displayed washability with minimal changes (R/R_0_ < 1.3) in resistance for 8 consecutive wash cycles while the CNT-coated cotton yarn was vulnerable and could barely satisfy such circumstances (Figure 3a) [147]. Alternatively, Jia et al. constructed a conductive core yarn wrapped with cotton fiber (roving) where a CNT yarn was introduced prior to the twisting zone. The multifunctional cotton fiber-wrapped CNT yarn retained its electrical properties without change in subsequent folding-releasing (~100 cycles) and washing (~5 cycles) (Figure 3b) [148].

The functional protein fibers (i.e., silk) are mostly produced by electrospinning (dry/wet/bio-mimetic) processes, which are accused of damaging the micro and nanostructures of the fiber. Thus, directly modified silkworm spinning is admired for keeping the inherent properties of the fiber intact. Wang et al. developed a functional native silk fiber via the continuous force-reeling and dip-coating technique (with CNT, Ag, and thermochromic paint) directly from Antheraea pernyi (A. pernyi) silkworms (known as Chinese Oak Tussah silkworms and having a similar primary structure to spider silk [149]). The functional fiber was highly stable and could withstand 48 h of washing without affecting the surface morphology (Figure 3c) [150]. Natural fiber in the form of liquid suspension is often prepared and utilized for improved electrochemical performance. Zhang et al. developed a thermally reduced graphene oxide (GO) cellulose composite paper-based pressure sensor (TRG-PS) from cotton pulp dispersion which displayed great cyclic stability (~8% changes in resistance for 300 bending-releasing) and washability up to 20 washing cycles with minimal resistance changes [151].

Fibrous materials are highly flexible to retain any shape as desired at the pre-stage of e-textiles development. Distinctive fiber architecture often offers better performance than regular configuration. A recent study reported a 3D helical fiber-shaped sensor with improved sensing performance (<1% detection limit), superior stability (no obvious change in >20,000 stretching cycles), and washability (no decay of electrical output in ten washing cycles) than regular fiber-shaped triboelectric nanogenerators (TENG). The helical fiber was obtained from the multiaxial winding of two core-shell braided fibers (Ag core in both fibers, whereas the shells were polytetrafluoroethylene-PTFE and nylon, respectively) followed by alternative winding on a stretchable fiber substrate (Figure 3d) [152]. The Helical fiber produced in a different but facile way, that is, pre-stretched (100–400%) polyurethane (PU) fiber with adhered copper fiber wrapped with glue, also showed satisfactory durability (stable against 500 stretching cycles and 100 min ultrasonic washing (Figure 3e) [153].

Fibers of all categories in the form of aqueous suspension synchronized with nanomaterials are of great interest and are produced through electrospinning, printing, and other solution-based methods for the development of e-textiles with long-lasting stability and durability. Liao et al. developed large-scale continuous fiber (~1500 km) lithium-ion batteries using the solution-extrusion method that displayed excellent stability (withstands up to 10,000 bending cycles with negligible decay) and durability (<10% loss of capacity) against different hostile events, i.e., water immersion, heavy pressure, washing, and hammer strike (Figure 3f) [154]. Conductive fiber materials are the fundamental building block of wearable e-textiles but are usually converted into the shape of yarn (continuous length) to enhance cohesion between them and make them suitable for subsequent transformation as required.

### 2.2. Yarn Shaped Durable E-Textiles

In general, yarn is a continuous assembly of fibers or filaments twisted/bonded together for improved mechanical properties, i.e., strength, flexibility, etc. Electronically active yarn can be constructed in different ways, i.e., by converting conductive fibers/filaments into yarn, imparting functionality at the yarn stage, and synthetic spinning of polymeric solution with conductive filler. The conductive yarn plays an important role in the architecture of the wearable system by interconnecting different units within the system and facilitates the fabrication of mass-scale electronic devices in the form of fabric or garments. The conductive yarn must be robust enough to withstand different physical, chemical, mechanical, and other hostile stimuli involved in daily use. The combination of nanomaterials at the yarn level expedites functionality-induced performance enhancement because of the increased contact surface area.

**Figure 4:**
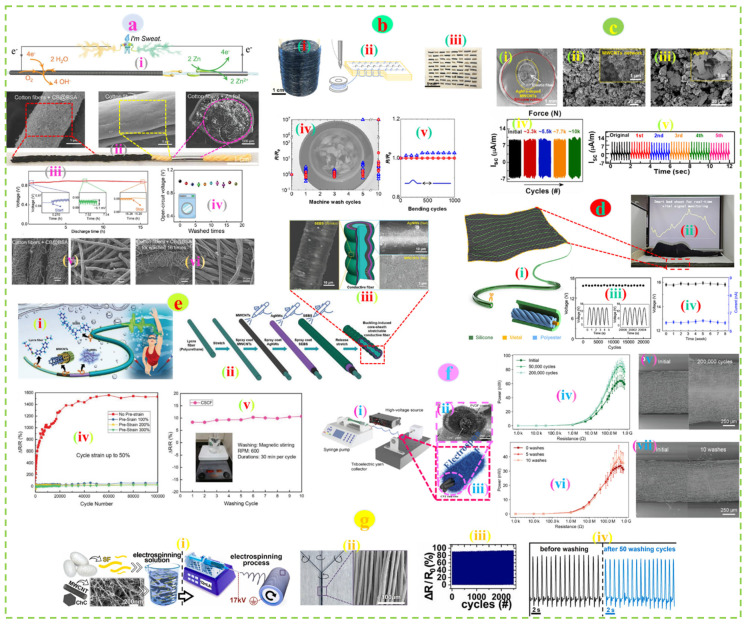
(**a**) (i) Schematic of the working principle of yarn-based sweat-activated battery, (ii) optical image of the substrate along with surface morphologies of different sections, (iii,iv) durability against cyclic operations and repeated washing cycles, (v,vi) surface morphology before and after 16 washing cycles. Reproduced with permission [156] Copyright 2022, Wiley. (**b**) (i) Roll (70 m) of conductive cellulose yarn, Schematic of the machine-sewn stitches (ii), and thermoelectric generator with 40 out-of-plane thermocouples (iii), (iv) resistance change after 10 machine washing and (v) 1000 bending cycles. Reproduced with permission [157] Copyright 2020, American Chemical Society. (**c**) (i) SEM images of the stretchable multifunctional fiber sensor, (ii,iii) Morphology of the MWCNTs-coated and AgNFs-doped-MWCNTs-coated fiber, and (iv,v) Current output (ISC) under repeated operations and washing. Reproduced with permission [159] Copyright 2022, Elsevier. (**d**) (i) Schematic of the sensing unit prepared by weaving functional yarn onto a black textile substrate, (ii) real-time sleep monitoring, and (iii) cyclic stability and washability (iv). Reproduced with permission [160] Copyright 2020, Elsevier. (**e**) (i) Schematic of the core-sheath yarn production process, (ii) Underwater sensing application, (iii) SEM images of the outer and inner layer of the yarn, (iv,v) Change of resistance under 50% cyclic strain and ten washing cycles. Reproduced with permission [162] Copyright 2019, Wiley. (**f**) (i) Schematic of triboelectric yarn production, (ii) SEM image of the yarn cross-section, (iii) CNT yarn core, (iv,v) Power output of the yarn upon 200,000 tapping cycles and changes in the surface morphology, (vi,vii) Wash durability and corresponding surface morphology of the yarn after repeated washing. Reproduced with permission [163] Copyright 2021, American Chemical Society. (**g**) Schematic of the electrospinning process (i), SEM image of the electrospun yarn (ii), cyclic stability (iii), and wash durability (iv) of the yarn. Reproduced with permission [164] Copyright 2021, Elsevier.

Gunawardhana et al. developed wearable triboelectric nanogenerators (TENGs) made of textiles (fabric made of Ag-coated nylon yarn) with differently coated triboelectric material (Polydimethylsiloxane-PDMS). It was observed that yarn-coated TENG outperforms other TENGs (i.e., screen printed and dip-coated fabric made of the same conductive yarn) in output due to higher triboelectric contact surface area. The electrical output of the yarn-coated TENG (i.e., open circuit voltage (V_OC_) ~ 34.5 V, short circuit current (I_SC_) ~ 60 nA, short circuit charge (Q_SC_) ~ 12 nC) was superior to that of other TENGs (screen printed; V_OC_ ~ 17.3 V, I_SC_ ~ 43 nA, Q_SC_ ~ 5 nC and dip-coated; V_OC_ ~ 4.9 V, I_SC_ ~ 11 nA, Q_SC_ ~ 2 nC) and showed better cyclic stability up to 3000 contact separation cycles [155]. Xiao et al. developed cotton yarn-based sweat-activated batteries (CYSAB) by drop coating black carbon (cathode, 4 cm), a bare portion (salt bridge, 0.5 cm), and subsequently wrapped with Zn foil (anode, 1.0 cm) of the same pristine cotton yarn. The device could withstand 2000 bending cycles and 16 washing cycles of 10 min each without a significant change in voltage output of the battery activated with 100 mL of salt solution (NaCl) (Figure 4a). The higher durability of the device was further verified by the unaffected surface morphology of the cathode portion against washing [156]. Electroactive regenerated cellulose yarn produced via roll-to-roll coating with poly(3,4-ethylenedioxythiophene):poly(styrene sulfonate) (PEDOT: PSS)/Ethylene glycol (EG) showed high conductivity (36 Scm^−1^) and durability. A thermoelectric energy harvester was designed by sewing the electronic yarn into a multilayered fabric. No resistance changes were observed for the device after repeated bending (1000 cycles) and machine washing (insignificant changes in the first five cycles, while further washing (<10) leads to notable changes) (Figure 4b) [157].

The core-sheath yarn structure holds great promise toward durability by combining nanoparticles in the core securely and preventing it from decay. Zeng et al. developed a highly durable wearable strain sensor based on a spandex dip-coated CNT core and cotton fiber sheath yarn. The sensor showed promising stability under 20% cyclic stress and ultrasonic washability (<5% deviation in resistance, five cycles) against water, acid, and alkali solution [158]. The self-powered sensor made of commercially available nylon/spandex yarn dip-coated with multi-walled carbon nanotubes (MWCNT) followed by spray coating with silver nanoflakes (AgNFs) and covered with silicone rubber showed good durability (~10,000 cycles of repeated operations) and washability (no significant decrease in performance against five repeated washing cycles) (Figure 4c) [159]. Zhou et al. demonstrated a polyester yarn twisted around a steel rod (10 µm dia.) covered with ultrathin silicon and weaved into a back textile substrate with the serpentine structure for sleep monitoring. The substrate was consistent under the repetitive pressure test up to 20,000 cycles and with insignificant variation in the electrical output after 8 weeks (20 min per week) of repetitive washing (Figure 4d) [160]. In the case of core-sheath yarn, where the conductive fiber is wrapped around a textile core, the twist count (number of twists per inch/cm) also plays an important role in enhancing conductivity and robustness. Higher twist counts (over twisting) tend to exert more stability in larger deformation and repeated washing actions [161].

Pre-stretching of the yarn (in case of stretchable substrate) prior to nanomaterial incorporation leads to the formation of a wrinkled surface, which allows the electroconductive properties to be more stable against mechanical deformation by a gradual release of the surface wrinkles upon stretching. Zhang et al. developed an underwater wireless charging patch made of pre-stretched polyurethane filament spray-coated with multi-walled carbon nanotubes (MWCNT), silver nanowire (AgNW), and styrene-(ethylene-butylene)-styrene (SEB), respectively. The device could withstand more than 100,000 stretching cycles under 50% strain and displayed good washability (up to ten cycles without significant resistance change) (Figure 4e) [162].

Electrospinning is widely being used for yarn-based washable e-textile development, which enables nanomaterial integration at the molecular level in the form of polymeric suspension (which contains both substrate and nanoparticles) spun into a continuous filament directly or the spinning of functional nanofiber around a conductive filament. A unique triboelectric yarn was manufactured via electrospinning of Poly(vinylidene fluoride) (PVDF) nanofiber around a CNT filament. The device showed phenomenal stability (~200,000 fatigue cycles) without a decrease in RMS (root mean square) power output; instead, a 33% increase in energy harvesting capability was observed with a peak power density of 20.7 μW cm^−2^. Furthermore, the yarn could withstand ten repeated washing cycles without a significant change in RMS power output. The slight resistance change observed in between five and ten washing cycles may be due to the small amount of water residue inside or slight damage due to washing (Figure 4f). However, the morphological analysis of the yarn after repeated tapping and washing showed no significant damage, apart from slight tearing of the PVDF fiber surface while the core was completely intact [163]. Medeiros et al. developed omniphobic silk-based coils (OSCs) made of electrospun yarn composed of silk fibroin, multi-walled carbon nanotubes (MWCNTs), and chitin carbon (ChCs) to power the wearable electronics remotely via magnetic resonance coupling. The device possessed great stability upon the repeated strain of 100% for 2500 cycles without a significant drop in performance. Furthermore, no performance degradation was observed even after 50 washing cycles (Figure 4g) [164].

Different yarn-shaped e-textiles and their endurance properties are presented in Table 1.

### 2.3. Fabric Shaped Durable E-Textiles

Fabric is the final phase of the textile hierarchy that enables mass-scale development of the e-textile component by either integrating it as an individual functional unit in the clothing or converting it into a complete wearable garment. Washable electronic fabrics can be obtained in many ways, such as by knitting or weaving the electroconductive yarn, by electrospinning an electronic nanofiber mat/film (nonwoven), or by direct incorporation of nanomaterials with them, etc. Satharasinghe et al. revealed that the washability assessment of photodiode-embedded yarns in both the e-yarn and the fabric form showed distinctive performance. For the e-yarn, the first failure was observed after 5 washing cycles and only 20% of them survived 25 washing cycles, while the fabric remained unaffected up to 15 cycles and 60% of them fully functioned after 25 cycles [176]. The e-yarns in the fabric form performed much better than in the yarn form and can be ascribed to the structural stability and compactness offered by the woven fabric.

The type, structure, and composition of the fabric affect not only the mechanical performance but also its operational longevity when combined with nanoparticles. Salavagione et al. demonstrated that different types of woven fabrics (regenerated cellulose, cotton, nylon, polyester, acrylic, and wool) have variant washability when coated with graphene/elastomer composite ink via hand printing. Although all samples showed stable performance (no change in resistance) against repeated folding (1000 cycles), in the case of washing, surprisingly, nylon and acrylic fabric had superiority (retained their initial resistance even after ten machine wash cycles) over others (significant loss of resistance) [177]. In a different study, polyester fabrics of different architectures, i.e., knit, woven, and nonwoven, demonstrated variable washing performance when coated with silver ink through the inkjet printing process. The woven fabric showed superior wash durability (insignificant resistance change after 15 machine washing cycles), while the knit fabric’s resistance doubled (>1 kΩ) after the same amount of wash cycles and 50 times higher resistance (2.3 Ω to >100 Ω) was observed for the nonwoven fabric only after a single wash. The poor resistance to washing of the nonwoven fabric may be ascribed to the looseness of the structure. Compact nonporous fabric structures (i.e., woven and knit) ensured better integration of conductive ink in the inkjet printing process, leading to better durability [178].

**Figure 5:**
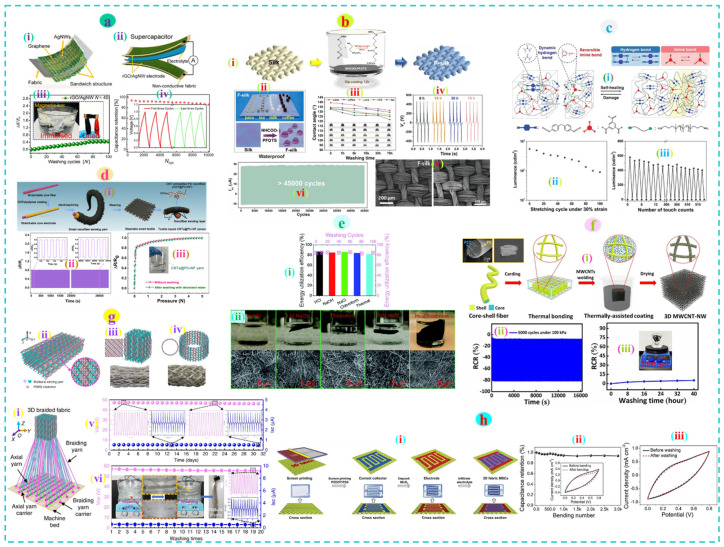
(**a**) (i) Schematic of the rGO/AgNw coated fabric and (ii) wearable supercapacitor, (iii) Change of resistance against repeated washing (100 times), (iv) Cyclic stability under 10,000 charge-discharge cycles. Reproduced with permission [179] Copyright 2021, American Chemical Society. (**b**) (i) Schematic of the self-cleaning functionalized silk production process, (ii) Waterproof properties against various liquids, (iii,iv) water contact angle and output voltage after 70 h of washing, (v) Surface morphologies of the substrate before and after washing, (vi) Stability of the TENG device upon repeated contact-separation cycles. Reproduced with permission [180] Copyright 2022, Elsevier. (**c**) (i) Schematic of the self-healing mechanism of the developed water-assisted self-healing polymer (WASHP), (ii) stability, and (iii) reproducibility of the WASHP-based light-emitting touch-responsive device upon cyclic stretching and cyclic switching, respectively. Reproduced with permission [181] Copyright 2021, Wiley. (**d**) (i) Fabrication of the wearable e-textiles pressure sensor, (ii) operational stability of the pressure sensor, and (iii) wash durability. Reproduced with permission [182] Copyright 2020, Elsevier. (**e**) (i) Energy utilization efficiency of the photothermal nonwoven fabric after washing and exposure to harsh environments, (ii) Optical and SEM images of the substrate after exposure to different harsh environments. Reproduced with permission [184] Copyright 2018, Royal Society of Chemistry. (**f**) (i) Schematic of the 3D nonwoven fabric-based piezoresistive sensor fabrication process, (ii) relative resistance change against 5000 compression/release cycles, and (iii) 40 h of washing. Reproduced with permission [185] Copyright 2021, Elsevier. (**g**) (i) Schematic of the 3D braided technology (ii–iv) Rectangular, Square and Toroidal shaped 3D TENG, respectively, (v,vi) Output voltage for prolonged operations (one month) and washability for 20 cycles. Reproduced with permission [186] Copyright 2020, Nature. (**h**) (i) Fabrication of 3D fabric-based micro supercapacitor, (ii) capacitance retention of the device over 3000 bending cycles, (iii) cyclic voltammetry curve of the supercapacitor before and after washing. Reproduced with permission [187] Copyright 2021, Wiley.

Kim et al. prepared a wearable supercapacitor made of supersonically sprayed cotton fabric with reduced graphene oxide (rGO)/silver nanowires (AgNWs) that revealed long-term cyclic stability (86% capacitance retention) under 10,000 operation cycles and exceptional aqueous wash durability (100 times) within acceptable relative resistance change (40% increase) up to 80 cycles and remained stable afterward (Figure 5a) [179]. Feng et al. developed a self-healing and self-cleaning triboelectric nanogenerator through the liquid-phase fluorination technique via dip-coating of silk and nylon fabric with urethane perfluorooctyl silane (NHCOO-PFOTS). The device showed superior durability. The water contact angle of various liquids (tea, coffee, juice, milk) experienced an insignificant decrease (5.02–8.21%) and the output voltage of the silk/nylon pair remained constant (maintained 96.77% of its original 465 V) even after 70 h of repeated washing. Furthermore, the device exhibited remarkable stability against 45,000 repeated contact/separation cycles with stable electrical output (power density 2.08 W.m^−2^ at 10 MΩ load) (Figure 5b). Such outstanding durability of the device was attributed to the strong bonding force between the hydroxyl and ethoxy groups of the fabric and the NHCOO-PFOTS molecules, respectively [180]. In a different study, He et al. developed a water-assisted self-healing polymer (WASHP) film based on covalent imine bonds crosslinked with hydrogen bonds with excellent mechanical flexibility (9050% strain) and self-healing capability (95%) in a shorter time (1 h). Later, the WASHP-based light-emitting touch-responsive device exhibited high stability (up to 72 cycles) under cyclic stretching at 30% strain and excellent reproducibility against cyclic switching (on/off) for 515 cycles under pressure (Figure 5c). The application of such self-healing polymers together with nanoparticles can be applied to textiles for designing highly flexible, durable, waterproof, wearable soft electronics [181].

Qi et al. developed a wearable e-textiles pressure sensor by plain weaving of CNT embedded electrospun nanofiber yarn. The device had superior stability with insignifcant resistance change against 10,000 operation cycles under 0.1 N pressure. Besides, no obvious change in electrical response was observed after 1 h of continuous water washing (Figure 3d) [182]. The functional nonwoven fabric made of electrospun cellulose/polyaniline (PANI) nanofiber showed excellent electromagnetic interference (EMI) shielding efficiency even under cyclic twisting (1000 times) with no decay (99.68% of the wave dissipated) and ultrasonic washing (99% of the incident EM wave attenuated) for 10 min. Morphological analysis of the fabric revealed substantial damage to the surface fiber even in a quick wash (10 min) indicating the vulnerability of such a porous nonwoven structure and ineffective integration of PANI molecules in the dip-coating process [183]. Jin et al. demonstrated that an electrospun nonwoven photothermal fabric made of nylon and carbon is capable of absorbing 94% solar spectrum with 83% solar energy utilization efficiency. The fabric was highly washable (100 hand wash cycles) and could withstand different harsh environments (Figure 3e) for a longer period (3 weeks) [184].

Tian et al. reported a wearable piezoresistive sensor from polyester (PET)/polyethylene (PE) fiber-based 3D nonwoven fabric coated with multi-walled carbon nanotubes (MWCNT). The sensor showed excellent operational stability against 5000 compression/release cycles with a constant relative resistance change (RCR) pattern. The initial slight drop in RCR may be attributed to slight plastic deformation of the fiber under pressure. Furthermore, the pressure sensor could withstand 40 h of vigorous washing with an acceptable change in RCR (8.4%) (Figure 5f) [185]. Dong et al. reported a 3D-shaped braided TENG via multiaxial winding of commercial silver-plated nylon yarn coated with PDMS. The 3D braided TENG structure displayed an improved electrical output than the traditional 2D TENG fabric due to its larger contact separation gap. In addition, the device showed long-term stability (one month of cyclic loading) and washability (20 times) without a significant decrease in electrical output (V_OC_, I_SC_) (Figure 5g) [186]. Li et al. developed a 3D fabric-based micro supercapacitor through screen printing of the poly(3,4-ethylenedioxythiophene):poly(styrene sulfonate) (PEDOT:PSS) interdigitated pattern followed by MnO2 deposition and electrolyte penetration. The excellent stability of the supercapacitor was confirmed by the higher capacitance retention (94%) after 3000 bending cycles. The device showed excellent durability, with no changes in the cyclic voltammetry (CV) curve before and after washing. Furthermore, the 3D fabric-based supercapacitor exhibited a remarkable areal capacitance (~135.4 mF cm^−2^) 3.5 times higher than that of the planar substrate (PET) based supercapacitor (Figure 5h) [187].

Different fabric-shaped e-textiles and their endurance properties are presented in Table 2.

## 3. Interconnections

**Figure 6:**
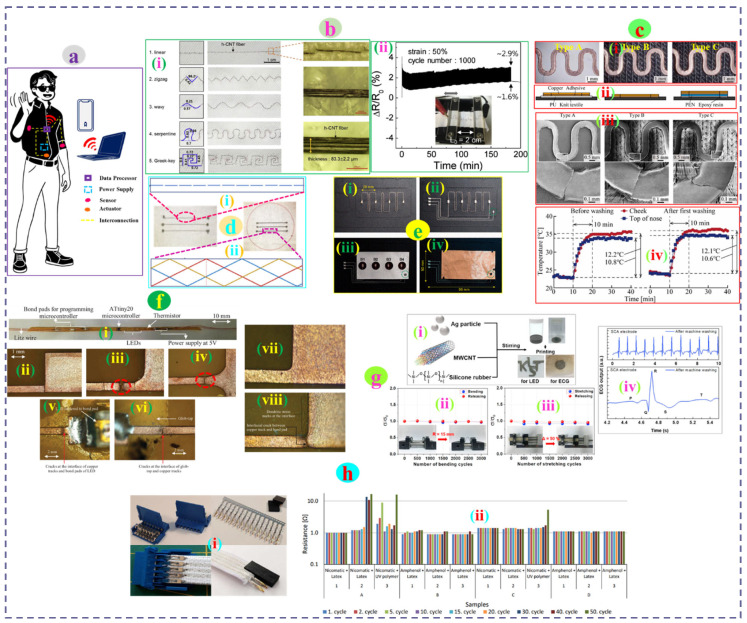
(**a**) Basic architecture of a wearable electronic textiles system embedded with different functional units interconnected together. (**b**) Different interconnection patterns made of hybrid CNT fibers (i) and (ii) cyclic stability of the serpentine pattern under 50% strain. Reproduced with permission [200] Copyright 2019, Elsevier. (**c**) (i) Optical images of the differently attached serpentine interconnect on the knit substrate, (ii) Cross-section and (iii) SEM images for each type of attachment upon cyclic deformation, (iv) variations in temperature before and after the first washing. Reproduced with permission [201] Copyright 2021, Institute of Physics. (**d**) Interconnection pattern made by single-line stitch sewing (i) and three-line stitch embroidery (ii). Reproduced with permission [202] Copyright 2020, Elsevier. (**e**) One-stop production of a four-button touch sensor. (i) Embroidery of the sensing pattern using Ag coated PA yarn, (ii) Embroidered metal composite yarn circuit for interconnections, (iii) placement of mesh spacer insulation layer, and (iv) attachment of upper conductive layer in the assembly by normal yarn satin stitch. Reproduced with permission [203] Copyright 2017, Sage Publications. (**f**) Different components attached to the filament using solder paste (i), post-bending failure of the copper tracks with no fillet (ii), filleted at the edge (iii), and center of the bond pad (iv), failure of the copper tracks upon bending without (v) and with glob-top encapsulation (vi), optical images of the circuit before (vii) and after washing (viii). Reproduced with permission [204] Copyright 2019, Wiley. (**g**) Schematic of the stretchable conductive adhesive (SCA) paste preparation (i), change of conductivity upon cyclic bending (ii) and stretching (iii), ECG output of SCAs as electrodes at machine washing (iv). Reproduced with permission [205] Copyright 2019, ACS publications. (**h**) (i) images of Amphenol and Nicomatic Crimp, (ii) resistance of different crimp interconnects after 50 cycles of washing. Reproduced with permission [206] Copyright 2021, IEEE.

The wearable electronic system may involve the integration of different multifunctional, stimuli-responsive electronic textile components interconnected with other circuit elements for operation (Figure 6a). Interconnections among different sensing, data acquisition, and processing units are very crucial to transmit information in the form of a data signal from one unit to another in synchronization with the actions of the wearer. Thus, interconnection lines must maintain optimal endurance against all physical, chemical, mechanical, and other stresses that may occur during operations without affecting their signal transmittance capability. Electroconductive fibers and yarns offer the optimum degree of flexibility required in wearable operations, in comparison to commercial metallic wires that are incompatible and may fail in repeated operations. Thus, conductive textile fibers/yarns are the best suit for wiring different components present in a wearable system, but they need to be robust enough to consistently serve their purpose throughout the lifespan of the wearable architecture without losing their functionality. The interconnections among different wearable components can be achieved via physical (soldering, conductive/nonconductive paste, crimp) or mechanical (embroidery, printing, sewing) bonding of the interconnects with the clothing/garment embedded into the complete wearable system. The physical methods are mostly adopted for interconnecting commercial rigid electronic components which involve a high welding temperature (soldering), may easily break under greater deformation (clamp), and may easily be affected by humidity and temperature (adhesive paste) [199], etc. These interconnection techniques are not suitable for designing flexible and comfortable wearable systems. On the other hand, the mechanical bonding, i.e., stitching of flexible yarn/filament-shaped interconnects via sewing or embroidery, ensures firm and reliable connections among the existing units of the wearable system. Such integration of interconnects is compatible with the textile-based wearable architecture and may not affect wearer comfort. However, the endurance of the transmission line is crucial, which depends on the type and durability of the conductive substrate adopted for interconnection, the integration method, and the pattern.

Eom et al. demonstrated highly conductive (1300 S/cm) and stretchable ionic liquid/poly (vinylidene fluoride-co-hexafluoropropylene) modified dry spun CNT fibers for interconnections in wearable e-textiles. A commercial sewing machine was used to design a variety of patterns on a fabric. The linear interconnection pattern showed the lowest electrical resistance (88.9 Ω) while an increasing trend was observed for more complex patterns, respectively. The CNT fiber showed optimal durability under 30 min of domestic washing with a slight loss of conductivity (1015 S/cm). The fiber interconnection with the serpentine pattern also displayed excellent cyclic stability under 50% strain for 1000 stretch-release cycles with only a 2.9% variation in relative resistance (Figure 6b) [200]. Koshi et al. demonstrated that serpentine interconnects with different laminated structures (Type A/B/C) exhibit a distinctive failure lifetime against consistent elongation. Type C interconnection was found to be the most durable (survived 224 cycles) against applied strain compared to other types of interconnects. Moreover, the cloth face mask built with type C interconnects showed optimum skin temperature monitoring performance even after eight washing cycles, beyond which multiple failures were observed (Figure 6c) [201]. Atakan et al. revealed the durability performance of the silver-plated polyamide yarn transmission lines on the cotton fabric produced by the sewing technique (single-line stitch) and the embroidery technique (three-line stitch). Martindale abrasion tests of the interconnection lines under both dry and wet conditions showed that the silver coating was more damaged in the wet states than in the dry medium. However, in terms of integration technique, embroidery transmission lines exhibited better electrical performance promoted by numerous interconnection points present in the embroidery network (Figure 6d) [202]. In a different study, one-stop production of multilayer structured four-button textile touch sensors was achieved via embroidery of a silver-coated polyamide yarn sensing pattern interconnected by embroidered metal composite yarn onto commercially metalized nylon fabric. Mesh spacer fabric was inserted to act as an insulation layer between the top layer (conductive fabric) and the bottom circuit layer. Both the embroidered sensing unit and the interconnections are expected to be highly durable against external impacts during operations, as they are securely held within the compact fabric assembly structure (Figure 6e) [203].

Komolafe et al. reported filament circuits containing LEDs and other components along with connection wires attached by using anisotropic conductive paste and stencil printed solder paste, respectively. The joint between the track and the bond pad was the main failure point in bending for both filleted and unfilleted filaments and could withstand similar bending cycles (62) before failure. However, glob-top encapsulation of the filaments significantly improved bending resistance and survived twice the number of cycles of the unencapsulated filaments. The filament circuits were later successfully incorporated inside a narrow pocket within the fabric during the weaving process to assess durability against repeated bending and washing. The encapsulated filaments embedded in the fabric survived 1500 repeated 90° bending cycles at a bending radius of 1 cm. The glob-top encapsulation resulted in improved durability but could not provide full protection for a prolonged duration, as the failure point migrates toward unencapsulated areas of the filaments. Therefore, encapsulation of the entire filament could provide a reliable performance output upon mechanical stress. The unencapsulated filament showed poor washability and after only five cycles interfacial crack and dendritic stress on the circuit were noticed. Moreover, the fully encapsulated filament with thermally molded Kapton within the fabric survived up to 45 domestic washing cycles (Figure 6f) [204]. Ko et al. reported a stretchable conductive adhesive (SCA) paste containing Ag particles, MWCNT, and silicon rubber to be utilized as printable interconnects in the joining of e-textile components. The printable interconnects were highly stable under larger stretch (120%) and exhibited no resistance changes when connecting LEDs to the battery. The higher stability of the adhesive specimen was further confirmed by repeated bending at a radius of 15 mm and stretching under 50% strain for 3000 cycles with an insignificant change of conductivity (σ/σ0) of 0.97 and 0.91, respectively. The SCA paste was applied to the PDMS and bandage to assess its durability against paste mixer and domestic washing, which revealed its excellent endurance with almost no change in electrical resistance. The excellent washability of the SCA electrodes was further confirmed by the unchanged ECG signal pattern even after washing (Figure 6g) [205]. Sima et al. evaluated the stability and durability of two commercial crimps for detachable interconnections of textile ribbons containing four conductive paths made of a hybrid thread containing PET, Ag, and Cu. The Amphenol crimp connectors showed superior stability under 50 domestic washing cycles compared to the Nicomatic crimp, which showed instability or deterioration of contact between the crimps and the conductive paths of the ribbon. The protective coating of UV polymer and latex near the ribbon connector had no influence on the durability of the interconnections after washing (Figure 6h) [206].

Interconnections are an integral part of the wearable e-textile assembly to satisfy the customer’s reliability in data acquisition and analysis. Thus, the interconnection lines must be flawless and exhibit the same degree of robustness as the whole wearable system. Flexible interconnect materials, connection patterns, and joining techniques all must be in synchronization to achieve the highest possible endurance throughout the entire life cycle of the wearable garments.

## 4. Durability Enhancement Strategies

Although different textile structures with variant incorporation techniques with a wide range of nanomaterial selection windows have been investigated, poor durability of the e-textiles is still challenging and may be linked with the mismatch of properties between the substrate and electroactive materials. Thus, to retain the functionality even in a hostile environment, the researcher often adopts different durability enhancement strategies to be employed in various stages, i.e., pre-treatment, during the process, post-treatment, etc. The following section of the article briefly describes numerous techniques aimed at the development of durable e-textiles.

### Surface Modification

**Figure 7:**
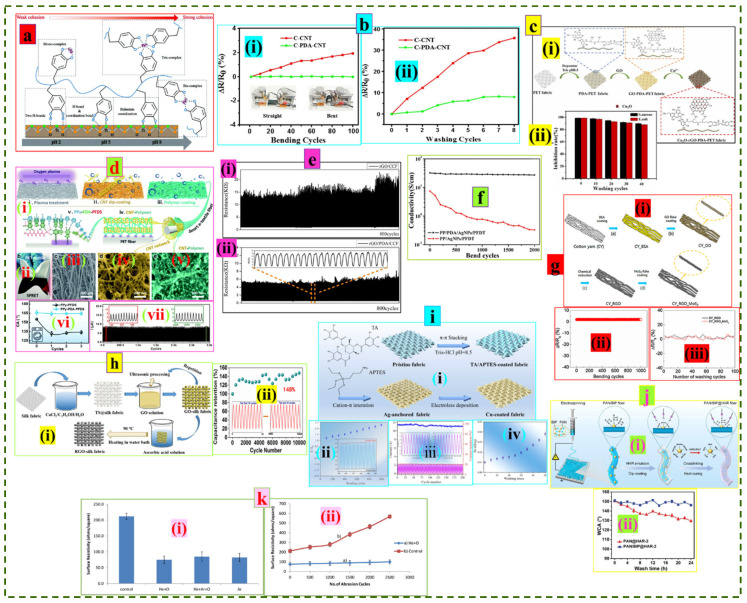
Different surface modification strategies. (**a**) pH-dependent chemistry and adhesion mechanism of polydopamine. Reproduced with permission [207] Copyright 2020, Royal Society of Chemistry. (**b**) (i,ii) Stability and washability of the PDA-modified CNT-coated cotton composite fabric. Reproduced with permission [208] Copyright 2019, Springer. (**c**) (i) Schematic of the PET fabric modification process with dopamine, and (ii) Antibacterial properties against different washing cycles. Reproduced with permission [209] Copyright 2022, Elsevier. (**d**) (i) Schematic of the self-protective and reproducible e-textile (SPRET), (ii) optical images of the substrate, (iii–v) SEM images of the SPRET, CNT network, and CNT polymer composite, respectively, (vi) washing assessment, and (vii) cyclic stability against 3000 loading/unloading cycles. Reproduced with permission [210] Copyright 2019, Royal Society of Chemistry. (**e**) Cyclic stability of the RGO-coated PDA-modified cotton fabric (i,ii). Reproduced with permission [211] Copyright 2020, Springer. (**f**) Changes in conductivity of the dopamine-modified nonwoven under 2000 bending cycles. Reproduced with permission [212] Copyright 2019, Elsevier. (**g**) (i) Schematic of the composite yarn production process pre-modified with bovine serum albumin (BSA), (ii) stability, and (iii) washability. Reproduced with permission [215] Copyright 2017, Elsevier. (**h**) (i) Schematic of the RGO/silk fabric fabrication process, (ii) Stability under cyclic operation cycles. Reproduced with permission [217] Copyright 2022, Elsevier. (i) (**i**) Schematic of the metal nanoparticles deposited PA6 fabric development process, (ii) relative resistance change upon repeated bending, (iii) current output stability, and (iv) washability test. Reproduced with permission [218] Copyright 2022, Elsevier. (**j**) (i) Schematic of the membrane production, surface modification, and functionalization process, (ii) Changes in water contact angle (WCA) after washing. Reproduced with permission [220] Copyright 2022, American Chemical Society. (**k**) (i) Surface resistivity of the PPy-coated polyester cotton blend fabric pre-treated with various plasma, and (ii) durability against repeated abrasion cycles. Reproduced with permission [222] Copyright 2020, Taylor & Francis.

The surface modification of the substrate is usually done in the pre-treatment stage prior to nanomaterial integration, and it is found to be an effective way of introducing a functional chemical group on the non-polar textile’s surface to accelerate chemical bonding and adhesion in between the substrate and nanoparticles for improved endurance. The mussel inspired bio-protein, i.e., dopamine, is known to be an efficient material for prompting interfacial bonding in textile functionalization by mimicking the adhesive behavior of the natural mussel with minimal environmental impact. The pH plays an important role in dopamine interaction and adhesion properties with the formation of different complexes, i.e., mono, bis, and tris complexes with Fe^3+^ (Figure 7a) [207]. Polydopamine (PDA) obtained through in situ polymerization (pH ~ 8.5) of dopamine is enriched with a catechol and amine functional group which facilitates strong adhesion of the coating materials with the textiles. Sadi et al. developed a highly durable multifunctional cotton fabric via polydopamine-templated dip-coating of single-walled carbon nanotubes (SWCNTs). The modified cotton fabric showed a greater adhesion behavior, which resulted in higher conductivity (41.5 Ω/Sq.) and performed consistently upon repeated bending (no change, ΔR/R_0_ (%) ~ 0%) and washing (slight change, ΔR/R_0_ (%) < 10%) while the fabric without PDA was vulnerable to such actions (Figure 7b) [208]. In another study, an antibacterial fabric made of PDA-inspired polyethylene terephthalate (PET) fabric coated with RGO and Cu_2_O showed higher conductivity (~2.7 times) than the original substrate. The fabric was highly washable and could withstand repeated washing with optimal antibacterial performance (90% for *S. aureus* and 88% for *E. coli*) even after 40 cycles (Figure 7c) [209]. A self-protective and reproducible e-textile (SPRET) was constructed by hierarchical ‘steels-concrete’ construction with a multifunctional polypyrrole (PPy)-polydopamine (PDA)- perfluorodecyltrlethoxysilane (PFDS) polymer ‘concrete’ layer on CNT ‘steel’-coated PET substrates. The promising machine washability of SPRET (three cycles, no change in contact angle; CA > 150°) over CCET (CNT-coated e-textiles) coated with PPy-PFDS (after only one cycle, CA decreased sharply) can be attributed to the presence of PDA-induced strong adhesion between polymer concrete and CCET. Additionally, no visible degradation of the output current in 3000 compressive cycles confirmed the higher stability of the composite (Figure 7d) [210]. Liu et al. demonstrated that the PDA layer between the cotton substrate and reduced graphene oxide (rGO) promotes greater durability under cyclic loading (800 cycles). The rGO/PDA/carbonized cotton fabric showed more stable and durable behavior with no notable resistance damage because of the improved connections between the fabric surface and the rGO. Alternatively, in the absence of PDA, the strain-induced microcracks in the rGO layer can barely merge after strain release, resulting in more unstable and larger variations of resistance (Figure 7e) [211]. Gao et al. developed an EMI shielding textile via the dip coating of PDA functionalized polypropylene (PP) nonwoven fabric with silver nanoparticles (AgNPs). The presence of PDA in the composite fabric greatly enhances the stability of the surface conductivity with almost no change upon repeated bending (2000 cycles), whereas no PDA modification of the same substrate leads to the sharp decline of conductivity (7.4 S/cm to 0.33 S/cm). Additionally, the composite showed excellent aqueous (acid solution, pH = 1) durability by retaining 84% of its shielding efficiency after 6 h of immersion (Figure 7f) [212].

Bovine serum albumin (BSA), an amphiphilic bioprotein often termed ‘universal glue’, offers a promising pathway to enhance the durability of e-textiles by attaching different organic and inorganic materials to the substrate via hydrophobic and hydrophilic interactions [213]. Zhou et al. developed a fiber-shaped supercapacitor by coating BSA templated cotton thread with MWCNTs/graphene hydrogel suspension. The device showed outstanding cyclic durability during 8000 operation cycles with 95.51% capacitance retention ability [214]. A flexible yarn-shaped gas sensor made of BSA-modified cotton yarn coated with RGO/MoS2 displayed excellent stability (normalized resistance change, ΔR/R_0_ ~ 2.1%, for 1000 bending cycles) and washability (100 times, with only 6% resistance loss) due to the strong molecular interactions of the coating materials with the yarn (Figure 7g) [215]. Yu et al. demonstrated that the BSA-induced surface modification turns ultra-high molecular weight polyethylene (UHMWPE) fiber into an adhesive platform for efficient assembly of MXene via the electrostatic wrapping technique. The strong interfacial bonding between UHMWPE fiber and MXene initiated by BSA resulted in improved mechanical properties (64% increase in interfacial shear strength compared to pristine fiber) but did not affect inherent fiber properties. The fiber morphology was found to be intact even after 30 min of ultrasonication with consistent electrical output (current, µA) [216].

Instead of using a common cross-linking agent, Cai et al. proposed a ternary solvent (CaCl_2_/C_2_H_5_/2H_5_OH/H_2_O) surface modification technique of silk fabric before coating it with RGO to develop flexible supercapacitor electrodes. Pretreatment with ternary solvent significantly improved the RGO loading on the substrate and showed 1.69 times lower sheet resistance (Ω) than without ternary solvent modification. In addition, the electrode maintained excellent capacitance retention (148%) at 10,000 charge-discharge cycles (Figure 7h) [217]. Du et al. constructed a highly durable wearable heater from Tannic acid (TA)/Aminopropyltriethoxysilane (APTES) functionalized Polyamide 6 (PA6) woven fabric coated with electrolessly deposited copper (Cu). The device could withstand 1000 stretch-release cycles with greater stability (R/R_0_ ~ 2.5) and retained a 95% current interception rate for 200 repeated operations (voltage on-off). Furthermore, the superior washability (50 times, nominal increase in resistance, 0.01 Ω/Sq. to 0.0375 Ω/Sq) of the device can be attributed to increased adhesion due to the presence of the TA/APTES anchor layer between the fabric surface and Cu molecules (Figure 7i) [218].

The homogeneous suspension of nanomaterials and modifiers is often prepared and preferred to facilitate mass-scale e-textile fabrication with improved durability rather than separately modifying the substrate in the pretreatment stage followed by functional coatings. Jiang et al. developed a polyamide fabric electrode via screen-printing of homogeneous thermoplastic polyurethane (TPU)/multi-walled carbon nanotube (MWCNT) ink. The excellent stability (0.8% resistance after 1000 bending cycles) and washability (only 2.1% increase in resistance after 20 wash cycles) of the electrode are attributed to increased adhesion due to the abundance of carbonyl and amino functional groups present in the TPU polymer matrix, which initiates direct bonding of MWCNT molecules with the polyamide fabric surface [219]. Li et al. developed a waterproof and breathable membrane from electrospun polyacrylonitrile (PAN) and blocked isocyanate prepolymer (BIP) nonwoven fabric dip-coated with fluorine-free waterborne hydroxyl acrylic resin (HAR). Cross-linking of the BIP enables the long hydrocarbon chain of HAR to be firmly attached to the PAN surface, thus improving the durability. The water contact angle (WCA) of the functionalized BIP membrane remained unchanged after 24 h of washing and UV irradiation. On the contrary, the WCA of the membrane without BIP decreased significantly upon 24 h washing (150.6 to 129.9) and UV irradiation (147.5° to 132.9°) (Figure 7j) [220]. Zhu et al. developed a highly conductive woven fabric via dip-coating with a homogeneous composite suspension containing single-walled carbon nanotubes (SWCNTs) and biomass-derived glucaric acid/chitosan (GA-chitosan) organic salt. GA-chitosan acts as an organic solvent-free green cross-linking agent to facilitate the higher stability and durability of conductive textiles. The fabric exhibited constant electrical performance (maximum resistance change <13%) for 1000 bending cycles and stable washing behavior with a slight loss of conductivity (15.1 S/cm to 12.5 S/cm) after 20 cycles [221].

Plasma-induced surface modification of textile substrates can substantially enhance the adhesion of functional nanomaterials for higher conductivity and endurance. Deogaonkar (2020) demonstrated that dielectric barrier discharge-atmospheric pressure plasma (He, O, Ar) pretreatment of the polyester cotton blend fabric significantly improved the binding strength of the Polypyrrole (PPy) coating with the substrate, resulting in a higher conductivity (75 Ω/Sq.) than that of the untreated fabric (210 Ω/Sq.). The surface resistivity of the modified sample experienced a minor increase (38%, 75 Ω/Sq. to 99 Ω/Sq.) after 2500 abrasion cycles, while the unmodified fabric was not competent (168% increase, 210 Ω/Sq. to 568 Ω/Sq.) (Figure 7k) [222]. 

Table 3 summarizes different surface modification techniques toward durable e-textiles.

The encapsulation of the e-textiles is commonly adopted to elevate endurance behavior and is performed mainly in the post-treatment phase. The thin layer of different encapsulating material provides versatile protection for the conductive pattern to be firmly anchored to the substrate and remains unaffected or less affected by the mechanical stress involved in daily use. It has been well established that the encapsulation of e-textiles provides better performance in terms of stability, flexibility, durability, washability, etc.

**Figure 8:**
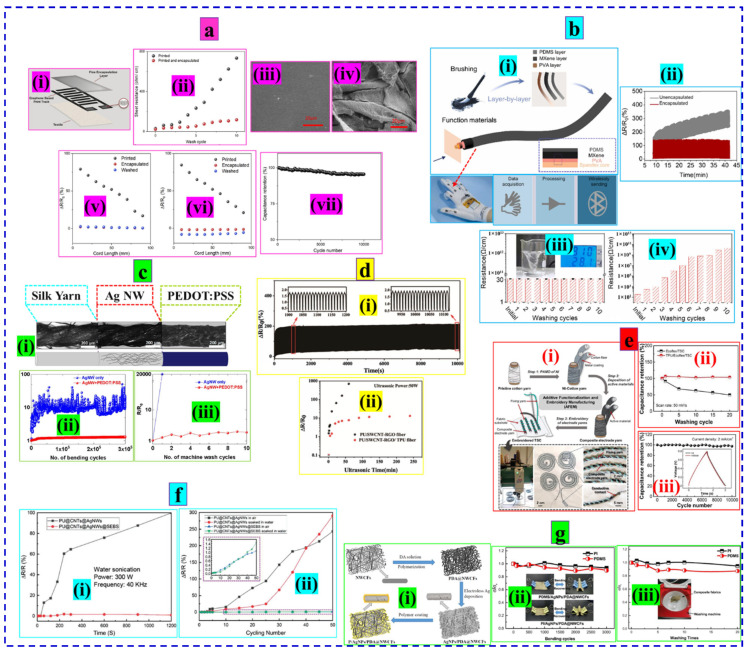
Different encapsulation strategies. (**a**) (i) Schematic of the encapsulated substrate, (ii) Change of resistance upon ten washing cycles, (iii,iv) SEM images of the encapsulated and unencapsulated fabric after washing, (v,vi) Variations in resistance upon repeated bending and compression in the forward direction, (vii) Capacitance retention under 10,000 operation cycles. Reproduced with permission [235] Copyright 2022, Cell Press. (**b**) (i) Schematic of the encapsulated fiber fabrication process, (ii) Stability under 500 stretch-release cycles, and (iii,iv) washability of the unencapsulated and encapsulated sample. Reproduced with permission [236] Copyright 2022, Elsevier. (**c**) (i) Schematic and optical images of the different sections of the functional silk yarn, (ii) Cyclic stability, and (iii) washability performance of the substrate. Reproduced with permission [237] Copyright 2020, American Chemical Society. (**d**) (i) Stability of the TPU encapsulated SWCNT-RGO/PU yarn under 1000 stretching/releasing, (ii) Durability against 250 h ultrasonic washing. Reproduced with permission [238] Copyright 2021, Springer. (**e**) (i) Schematic of the yarn-shaped supercapacitor with different (Eco flex/TPU) encapsulation, (ii) washability, and (iii) operation stability. Reproduced with permission [239] Copyright 2020, Wiley. (**f**) (i) Change in resistance of the core-sheath yarn with/without encapsulation under 50% cyclic strain in water and air, (ii) Underwater sonication. Reproduced with permission [162] Copyright 2019, Wiley. (**g**) (i) Schematic of the nonwoven fabric functionalization and encapsulation process, (ii) conductivity ration with 3000 bending cycles, and (iii) repeated washing. Reproduced with permission [240] Copyright 2021, Elsevier.

Islam et al. constructed highly conductive (49 Ω/cm) and durable e-textiles by screen printing cotton fabric with graphene ink followed by fine encapsulation of the conductive pattern with PU-based encapsulant (PE773). The thin PE773 layer ensures the graphene ink adheres firmly to the substrate surface and protects it from the different hostile stimuli involved in regular wash and wear. The electrical resistance of the encapsulated fabric maintained acceptable changes (~3.5 times increase, 49 Ω/cm to 118.0 Ω/cm) for ten home laundry cycles, while the bare printed fabric suffered a ten times increase in resistance (49 Ω/cm to 734.0 Ω/cm) for the same amount of washing. The original surface morphology of the encapsulated fabric was retained during wash cycles, but substantial damage and removal of the graphene flake were observed for the bared fabric. In addition, the encapsulated sample showed excellent repeatability in bending (forward direction) and compression (backward direction) compared to the unencapsulated sample. The excellent stability of the device (supercapacitor) was further confirmed by higher capacitance retention (95%) after 10,000 cycles of operation (charge-discharge) (Figure 8a) [235].

Duan et al. proposed a highly durable Polydimethylsiloxane (PDMS) encapsulated Spandex/Poly (vinyl alcohol) (PVA)/MXene (SPMP) intelligent fiber connected with a waterproof electronic system for the wireless monitoring of underwater hand gestures. The resistance of the encapsulated device remained almost unchanged for ten washing cycles, while the unencapsulated sample became nonconductive (about 1 GΩ) only after six washing cycles. The excellent underwater reliability of the PDMS coating was confirmed by no change in resistance for 1 h of water immersion compared to bare fiber (20% increase in resistance) for the same period. Furthermore, the encapsulated fiber showed excellent durability and maintained an unchanged resistance profile for 500 stretch-release cycles compared to the unencapsulated fiber, which could hardly withstand such cyclic deformations (Figure 8b) [236]. The silk yarn dip-coated with Ag nanowires (AgNWs) showed outstanding durability after being encapsulated with poly(3,4-ethylenedioxythiophene) polystyrene sulfonate (PEDOT: PSS). The conductive yarn was highly stable against repeated washing (ten cycles) with a slight increase (two times) in resistance, while the silk yarn with only the AgNW coating lost its resistance by four orders of magnitude and became highly resistive after two wash cycles. The PEDOT: PSS coating provided versatile protection for the AgNw layer attached to the yarn and resulted in greater durability with a slight change (<30%) of resistance for 300,000 bending cycles than the unencapsulated yarn that underwent a two-fold increase in resistance with an unstable output profile (Figure 8c) [237]. The Polyurethane (PU)/Reduced graphene oxide (RGO)-Single-walled carbon nanotube (SWCNT) core-sheath yarn sensor encapsulated with thermoplastic polyurethane (TPU) demonstrated excellent durability for potential application in strain-induced human motion monitoring. The sensor displayed a completely stable and repeatable relative resistance profile pattern after 1000 stretch/release cycles at 50% strain. At moderate strain (100%), the RGO/SWCNT conductive layer on the unencapsulated yarn cracked, while the morphology of the encapsulated yarn remained intact. Furthermore, the higher washability (slight change, ΔR/R_0_ ~ 10 for 190 min ultrasonic wash) of the encapsulated sensor compared to that of the unencapsulated yarn (poor wash durability, ΔR/R_0_ ~ 690 at 60 min) validates the need for encapsulation to retain the electrical functionalities of e-textiles for the long term (Figure 8d) [238].

Huang et al. reported a yarn-shaped textile-based supercapacitor (TSCs) integrated onto fabric via the embroidery technique followed by encapsulation with Ecoflex and TPU laminating. The Ecoflex-coated supercapacitors suffered the moderate loss of capacitance, but the lamination of TPU as an additional protection layer over Ecoflex-coated samples ensured superior washability with no obvious change in capacitance after 20 machine washing cycles. The encapsulated device could maintain 97% capacitance retention after 10,000 charge/discharge cycles and 9 h of underwater operations (1000 charge/discharge cycles). Additionally, the sample displayed remarkable stability (94% capacitance retention) when bending 4000 times (Figure 8e) [239]. In another study, a stretchable core-sheath Polyurethane (PU)/Muti-walled carbon nanotube (MWCNT)/Silver nanowire (AgNW) fiber encapsulated with styrene-(ethylene-butylene)-styrene (SEB) showed excellent durability under ultrasonication and maintained greater stability by retaining its actual electrical properties (slight change, ΔR/R_0_ (%) ~ 1.6%) for periodic operations (50 cycles) in water and air. The absence of an encapsulation layer resulted in a greater variation in resistance (ΔR/R_0_ ~ 300%) for the same level of underwater/air operations (Figure 8f) [162]. A superhydrophobic electromagnetic interference (EMI) shielding e-textile was developed by electroless deposition of silver nanoparticles (AgNPs) on a PDA-pretreated cotton nonwoven fabric (PDA@NWCFs). The encapsulation of the functional Ag coating was performed by forming a thin layer of polydimethylsiloxane (PDMS) or polyimide (PI) onto the fabric surface, which not only prevents oxidation and corrosion of AgNPs but also provides hydrophobicity. Both samples encapsulated with PDMS and PI showed excellent stability (~3000 bending cycles) and washability (20 times) with minimal deterioration of conductivity (Figure 8g) [240].

Table 4 summarizes different encapsulation strategies to develop highly durable e-textiles.

## 5. Wash Reality

A typical washing cycle involves mechanical rotation or agitation of e-textiles loaded inside a domestic laundry machine in an aqueous environment containing cleaning chemicals (detergent) and the ballistic workload for a designated period at a certain temperature followed by rinsing, drum spinning, and drying. Damages or loss of functionality of e-textiles in washing could be associated with the following stresses: mechanical action (drum rotation, agitation, spinning) induced stress, temperature-induced thermal stress, washing media (water, acid, alkali) induced stress, chemical stress (due to cleaning chemicals), and drying stress (in case of machine drying), etc. The degree of damage in washing greatly depends on the magnitude of these stresses involved in different built-in washing programs, namely cotton, silk, delicate, delicate short, express, and wool, present in commercially available household washing machines. Besides, researchers often follow laboratory washing techniques (in a container/beaker, stirring/ultrasonication) that are commonly used for the fastness assessment in the clothing industry. Although it is recommended to follow a specific wash protocol based on the composition (natural/synthetic) of the clothing substrate, retaining the functional properties of the e-textiles under any washing is very crucial for end-user reliability. It is expected that different washing procedures will have a distinctive impact on substrate properties, making the perception of the washability of e-textiles ambiguous among researchers. The limited number of standard washing protocols forced the researcher to adopt different available or customized methods with varying parameters to define the washability of their product.

Table 5 summarizes various washing protocols adopted by the researcher to determine the durability.

From Table 5, it can be concluded that different research groups explained washability in distinctive ways to confirm the washability of their e-textile products. It is noticeable that some groups tried to replicate the washing environment of the existing standards from relevant science fields, while others opted for the traditional technique (only water immersion, stirring) to verify their claim on durability. It is predicted that the e-textiles graded as highly durable by following traditional laboratory washing (beaker washing) might not show the same washing performance under intensified washing actions involved in a commercial washing machine. A recent study revealed that highly conductive cotton e-textiles (233.4 S/cm) could withstand ultrasonic water washing with a nominal change in electrical properties (ΔR/R_0_ < 5%) and their surface morphology was completely preserved. Alternatively, the same substrate showed greater resistance variations (ΔR/R_0_ ~ 13%) with substantial damage to the morphology in machine washing [258]. Therefore, it is very important to understand washing damage and the mechanism before making a conclusion about washability.

### 5.1. Washing Stresses

**Figure 9:**
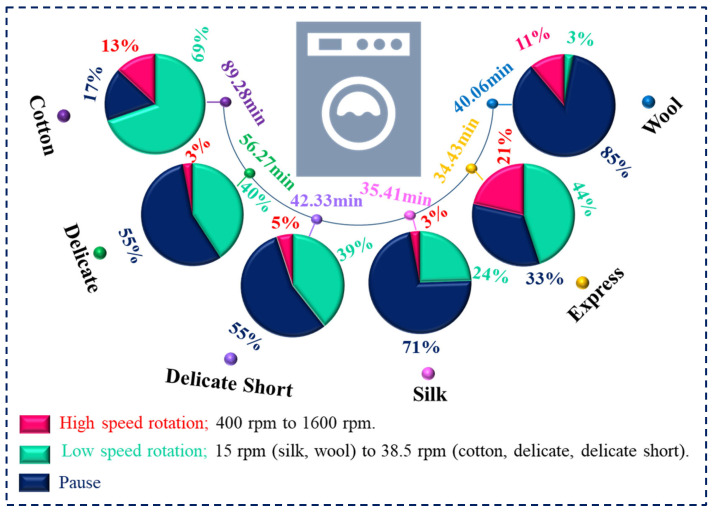
Different domestic laundry programs and their duration (min) configuration.

Washing, rinsing, and tumbling are the three main phases of a typical machine-washing process where the rotation speed (low, high, pause/rest) of the drum varies according to the chosen wash program (see Figure 9).

**Figure 10:**
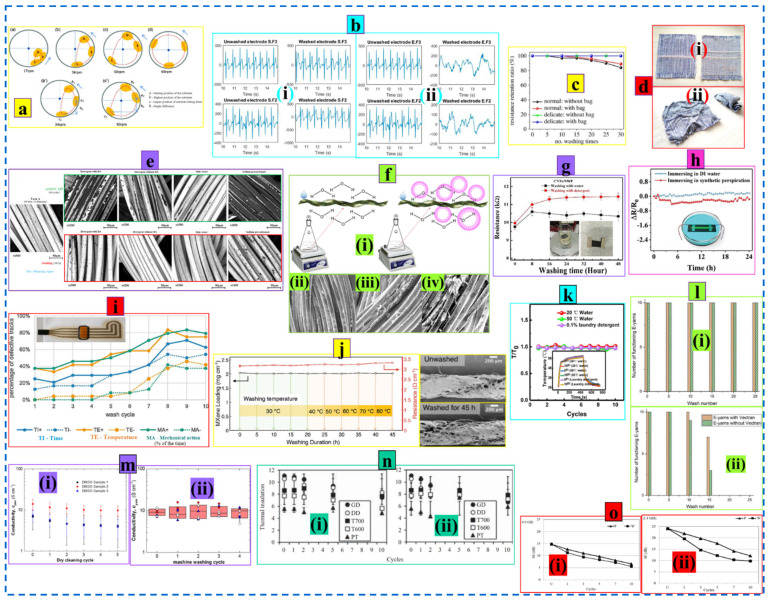
(**a**) Schematic of the fabric movement inside the drum at different rotation speeds. Reproduced with permission [260] Copyright 2020, SAGE Publications. (**b**) ECG measurement of the substrates, i.e., PET fabric/Cu (SF2) and PET fabric/Cu-Ni (SF3) after machine laundry following silk wash program (i) and express wash program (ii). Reproduced with permission [261] Copyright 2020, MDPI. (**c**) Machine washing performance of FCB with/without laundry bag. Reproduced with permission [263] Copyright 2014, The Royal Society. (**d**) Photographs of the e-textile substrate before (i) and after machine wash (ii). Reproduced with permission [258] Copyright 2019, Springer. (**e**) Surface morphology of the silver-plated textile electrodes upon different washing techniques under variant aqueous environments. Reproduced with permission [264] Copyright 2020, MDPI. (**f**) (i) Schematic of the water and detergent washing process, (ii) SEM images of the silver-coated polyamide yarn before washing, (iii) after 72 h of detergent washing, and (iv) only water washing. Reproduced with permission [265] Copyright 2019, Springer. (**g**) Water and detergent washing assessment of nonwoven fabric coated with CNT. Reproduced with permission [266] Copyright 2020, Elsevier. (**h**) E-textiles immersion in water and perspiration medium for 24 h. Reproduced with permission [267] Copyright 2022, Wiley. (**i**) Effect of time, temperature, and mechanical actions on the washability of the textile printed circuit board. Reproduced with permission [268] Copyright 2020, Taylor & Francis. (**j**) Influence of temperature on the washing performance of MXene-coated cotton yarn. Reproduced with permission [269] Copyright 2019, Wiley. (**k**) Effect of temperature underwater washing. Reproduced with permission [270] Copyright 2021, American Chemical Society. (**l**) Influence of after-machine wash drying techniques, (i) m/c wash—line dry, (ii) m/c wash—tumble dry. Reproduced with permission [271] Copyright 2020, MDPI. (**m**) Dry cleaning (i) and aqueous machine washing (ii) performance evaluation of conductive yarn. Reproduced with permission [254] Copyright 2017, American Chemical Society. (**n**) Changes in the thermal insulation of filled materials by repeated water washing (i) and dry cleaning (ii). Reproduced with permission [272] Copyright 2017, Emerald Publishing Limited. (**o**) Shielding efficiency of the silver-coated polyamide yarn upon dry and wet cleaning at 0.9 GHz (i) and 2.4 GHz (ii). Reproduced with permission [273] Copyright 2021, MDPI.

The mechanical stresses are considered the most influential damaging force involved in machine washing. The degree of drum speed (rpm) is very crucial in determining the washing effect on the e-textiles. Generally, pre-wash (soaking), main wash, and rinsing cycles of any built-in wash program involve resting and low/high-speed rotation of the drum followed by high-speed tumbling. Accelerometer-assisted real-time video analysis of the washed e-textiles revealed different mechanical actions and stress it had to experience inside the machine during domestic washing. Regardless of the duration of the washing program, it is the drum speed and stop time that significantly damage the e-textiles. During high-speed rotation (400 rpm), the substrate stuck to the drum wall, while at the lower speed (15–38.5 rpm), the sample fell repeatedly due to gravitational force, which may lead to significant physical damage to the substrate. The prolonged rest/pause period generated negligible mechanical stress and damage to the device during washing [259]. Bao et al. demonstrated the movement of wool fabric inside the washing drum and analyzed different rotation-induced damage to the fabric. It was observed that a low speed (34 rpm) rotation caused more damage (30% thread loss) compared to a high speed (66 rpm) rotation, where only 1% thread loss was reported. At any speed above the threshold value (66 rpm), the fabric adheres to the drum wall and rotates with it, introducing low impact and frictional forces between the fabric-wash load and the fabric-drum (Figure 10a) [260]. Zaman and co-workers adopted ‘silk’ and ‘express’ washing programs following the same standard (ISO 6330) to evaluate the washability of different textile-based ECG electrodes. The resistance of the copper-based electrodes increased moderately (10–15 times) when exposed to 50 cycles of the “silk” washing program but showed acceptable ECG signals. On the contrary, the same electrodes became completely non-conducive only after ten ‘express’ washing cycles and did not produce any ECG signal (Figure 10b) [261]. Interestingly, Ojuroye et al. in a different study concluded that higher rotation is more destructive than lower rotation when determining the washability of Polydimethylsiloxane (PDMS)-encapsulated e-textiles integrated capacitive sensory circuits by following the ISO 6330 standard. When washed with 800 rpm, the circuits lost their functionality after only a quick wash (15 min), while the same circuits survived 10–15 wash cycles with a lower speed (400 rpm) but longer wash duration (37–42 min). The instability and poor adhesion performance of PDMS during high-speed washing resulted in the detachment of the IC chip from the sensory unit. Therefore, it is very important to choose the right washing speed to evaluate the washing performance of e-textiles based on their composition and architectural assembly [262]. The laundry bag is commonly used in domestic washing, which may reduce the intensity of mechanical stress-induced damage encountered by the substrate circulating inside the machine drum. Q and XM demonstrated that the resistance retention ratio (%), i.e., the ratio of the number of knitted fabric circuit boards (FCB) that maintain electrical integrity to the total number of washed samples, increased by 89% when the FCBs were washed inside a laundry bag (Figure 10c) [263]. The ballistic wash load is often recommended in domestic washing, which ensures a uniform distribution of mechanical stress for efficient dirt/soil removal in case the washing substrates weigh less than the recommended capacity. However, the washing load could be responsible for the bending, folding, twisting, stretching, and other mechanical deformations of electronic textiles during washing (Figure 10d), which could significantly affect their functional behavior; therefore, the impact of the washing load cannot be ignored and should be studied extensively.

Apart from mechanical stress, the washing media (aqueous/non-aqueous liquid or solvent and cleaning chemical) induced stresses are also important and substantially affect the electroactivity of the conductive substrate upon washing. It is expected that the e-textiles will exhibit more stable performance upon water-mediated washing compared to detergent washing. Detergents are chemically active materials that initiate a pH-induced cleaning reaction to emulsify soils or dirt present in the substrate and may also remove functional coatings from the substrate surface by the simultaneous formation of micelles around the coating materials during washing. Gaubert and co-workers investigated the washability of three different silver-plated nylon yarn electrodes under various washing mediums that included detergent with and without a bleaching agent (BA), only tap water, and tap water with sodium percarbonate for standard machine washing (AATCC135) and prolonged immersion (30 h) without mechanical constraints. The machine washing of electrodes with BA-containing detergent greatly increased surface resistivity (R_30_/R_0_ ~ 93,295), while slight changes were observed, i.e., 2.4 and 0.9 for detergent without BA and water, respectively. Washing with sodium percarbonate had the most destructive impact on electrode conductivity (>10 MΩ) only after 20 cycles and could not be measured with the multimeter used. Alternatively, prolonged immersion (30 h) of electrodes in the same medium mentioned above had a much smaller impact on damaging conductive properties. The values of R_30_/R_0_ were found to be 1.47 and 0.88 for detergent with and without BA, 0.91 for water, and 1.13 for sodium percarbonate soaking. In addition, variations in electrical properties after washing were evident for the electrodes of different thicknesses (Figure 10e) [264]. Ismar et al. demonstrated that water immersion of silver-coated polyamide (PA) yarn caused more damage to the surface coating compared to detergent immersion for the same period (72 h) at 30 °C. The aggressive effect of water was more evident in the surface morphology of the yarn, where greater damage to the coating layer was observed for immersion in water than for immersion in detergent. Such interesting results may be attributed to the polar nature of H_2_O molecules or the lack of surfactants in the water solution and surfactant-induced homogeneous interactions of Ag particles in the detergent solution to lessen surface damage (Figure 10f) [265]. In a different study, contradictory washing behavior was observed for a pressure sensor made of a non-woven fabric coated with carbon nanotubes using the nano-soldering method, which showed better water washing performance than detergent washing for a period of 48 h (Figure 10g) [266]. However, in another study, screen-printed electronic textiles with Ag ink showed a more stable electrical behavior for prolonged immersion (24 h) in water compared to immersion with synthetic perspiration for the same period (Figure 10h) [267]. The water used to wash has a certain influence on the washing efficiency and damages the functionality of the substrate, especially when there is hardness or impurities present, so the quality of the water cannot be compromised during washing. Rotzler et al. investigated the effect of different washing parameters such as duration, temperature, and mechanical actions on the integrated conductive textile tracks in standard (ISO 6330) machine washing. The duration (15–35 min) of washing appeared to be the least influential factor in damaging the conductive tracks present in the circuit. The mechanical action and temperature were the most influential factors affecting circuit functionality, but for the textile printed circuit board (TEX-PCB) on polyester woven fabric, the effect of temperature surpassing mechanical stress in washing may be due to the mismatch of the coefficient of thermal expansion among different materials used in TEX-PCB construction (Figure 10i) [268]. Uzun et al. revealed that temperature-mediated washing caused nominal damage to the electrical resistance of cotton yarn coated with Ti_3_C_2_Tx MXene after 45 h of washing. At lower temperature (30 °C) washing, an insignificant resistance change was observed after 20 washing cycles and the resistance slightly increased by only 3% after 45 cycles when the temperature increased from 30 °C to 80 °C. The negligible effect of temperature on the electrical properties of the yarn upon washing is further confirmed by its unaffected surface morphology (Figure 10j) [269]. The wearable heater made of graphene-ink printed cotton fabric sustained 10 washing cycles in 0.1 wt.% detergent aqueous solution at different temperatures. The effect of the washing temperature on the heating performance of the device was found to be very minimal (1.38% and 1.46% variations in the heating temperature profile observed for washing at 20 °C and 50 °C, respectively) (Figure 10k) [270].

The washing temperature is sensitive to the substrate and usually varies based on the type of fiber they are made of and may influence the activation of the detergent and its effectiveness. In almost all commercially available domestic washing machines, the washing temperature can be configured from an ambient temperature to 90 °C. The after-wash drying conditions and temperature are very crucial as they may accelerate the damage of the e-textiles’ functionality and thus cannot be ignored. Hardy et al. investigated the effect of different after-wash drying techniques on the durability of electronically active yarn. All (five pieces) conductive yarns (copper wire wrapped over polyester yarn covered with knit braid) were embroidered on a t-shirt surface followed by domestic washing (ISO6330) and line or tumble drying. Lack of continuity, intermittent continuity, or higher resistance value (>5 Ω) of the E-yarn was considered a failure. All E-yarns survived 25 washing and line drying cycles with very nominal variations in average resistance from 0.8 ± 0.18 Ω (before wash) to 0.7 ± 0.08 Ω (after wash/line dry). However, almost none of the yarns could withstand 25 wash/tumble dry cycles, and failure started after 5 cycles, and beyond 15 cycles none of the yarns functioned. It was also observed that incorporating a carrier yarn (Vectran) with the E-yarn as a reinforcement could exert more durability, but devastating damage in washing/tumble drying was still maintained, while the washing/line drying sample was consistent (Figure 10l) [271].

The waterless or water-free non-aqueous washing technique holds great promise in reducing the water footprint. Ryan et al. investigated the effect of dry cleaning and machine washing on PEDOT: PSS dyed silk yarn. The yarn showed no change in electrical resistance after four cycles of domestic machine washing following the “hand wash” program, while dry cleaning with a common solvent (tetracholoroethylene) resulted in a decrease of conductivity by a factor of two (Figure 10m) [254]. Kim et al. demonstrated the effect of water washing and dry cleaning on the thermal insulation properties of natural (goose down, duck down) and synthetic (Thinsulate 700, Thinsulate 600, polyester) filled outdoor sportswear. The decrease in the thermal insulation rate for natural fillers was lower with water washing than with dry cleaning. Natural fillers showed high thermal insulation up to five cycles and decreased slightly up to ten cycles but maintained a much higher insulation value compared to dry cleaning. The thermal insulation of synthetic filler materials also decreased after water washing, but the change was much less than that of natural materials. The resistance to laundry was outstanding for synthetic filler, but the loss of thermal insulation property was dominant for dry cleaning compared to water washing (Figure 10n) [272]. Interestingly, in a different study, Pusic et al. revealed that the EMI shielding efficiency (SE) of the conductive knit fabric made of silver-coated polyamide yarn was more sensitive to wet (water) cleaning (ISO 3175-3) than to dry (perchloroethylene) cleaning (ISO 3175-2). At a frequency of 0.9 GHz, the fabric showed a linear and parallel decline in SE for the dry and wet cleaning techniques, where the largest difference was observed in the third cycle and continued until the tenth cycle. The loss of SE due to wet cleaning was more obvious than dry cleaning in this case. At a frequency of 2.4 GHz, better preservation of SE was observed for dry cleaning than for water washing (Figure 10o) [273].

From the above discussion, it can be concluded that, depending on the type of substrate to be washed either in a dry or wet medium, the intensity of the washing damage varies. It is obvious that not all textile substrates are suitable for all wash strategies in different media. Therefore, the proper selection of the substrate, washing techniques, drying procedure, assessment criteria, and all other influential parameters must be in sync to understand and describe washability in the best way. The limited number of standardized washing and assessment protocols not only forces researchers to rely on different ideas to describe durability but also hinders the reliability of e-textiles.

### 5.2. Standardized Protocols

Durability can be termed as the ability of electronic textile components to maintain their functionality completely intact or to be affected less without compromising the comfort of the wearer when exposed to any harsh environment involving a variety of physical, chemical, and mechanical stresses. Therefore, the washability of the e-textiles is measured in the form of a relative change in their electrical output, i.e., resistance, capacitance, conductivity, voltage, current, and other performance indicators, which are attributed to changes in contact properties of nanomaterials inside or at the interface of the substrate. So far, researchers have followed different alternative standards from other fields, laboratory-scale techniques, and sometimes personally customized operations to evaluate the washability of electroactive textile components due to the limited number of standard protocols specially designed for the e-textile systems. It is undeniable that substantial progress has been achieved in recent years towards highly durable electronic textiles, but mostly from a laboratory perspective, not on a large scale. Therefore, large-scale production along with optimum endurance performance will expedite market readiness and adaptability. This is where more standards will play a great role through proper improvisation of the current achievements toward customer-reliable e-textile products. Different international institutions such as AATCC, ISO, ASTM, IEC, IPC, and others are relentlessly working to develop standards for the durability assessment protocol of electronic textiles, and some of them have even come up with a draft of their proposed standards, but they have yet to be finalized and approved. The following table summarizes different existing and upcoming standards related to e-textiles that could be beneficial to the research community working in a similar area.

Table 6 summarizes different existing and upcoming standards specialized for wearable electronic textiles.

So far, several methods have been proposed regarding the terminology and resistance measurement of e-textiles. However, only a few already published methods are found to be dedicated to the evaluation of e-textiles’ durability under different conditions, but surprisingly, they have never been adopted by researchers to date. Besides, different standards specialized for the assessment of washability of electronic textiles are currently under development and are expected to be published shortly.

## 6. Conclusions and Prospects

**Figure 11:**
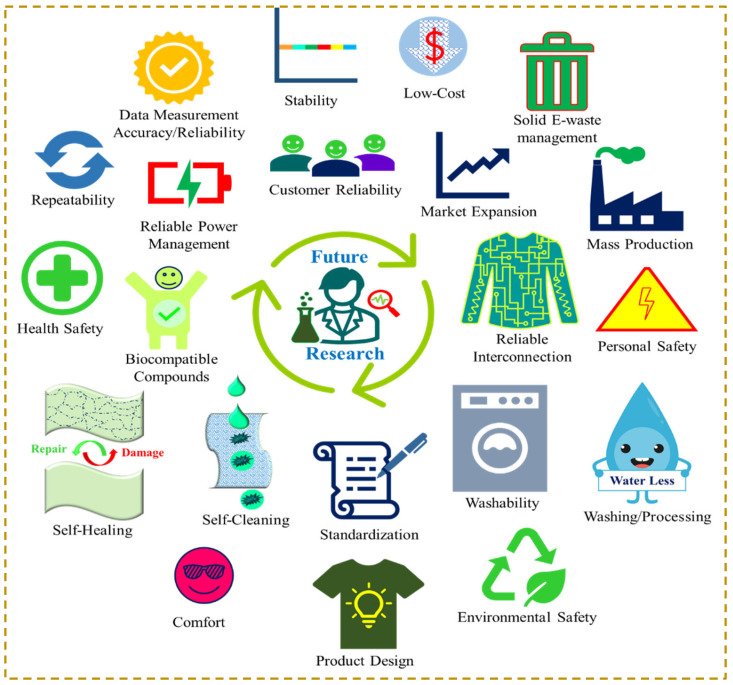
Futuristic highly robust and reliable e-textiles for long-term wearable electronic applications.

The wearable electronic textile utilizes different action-driven signals in measurable quantities with exciting possibilities in versatile areas along with personalized algorithms. Flexible electronic textiles are of great interest due to their ease of use, comfort, and compatibility at the user level. As discussed in the preceding sections, it is obvious that remarkable advances have been achieved in all possible aspects, from material selection to end-user-reliant, durable e-textile product design. Researchers have explored different architectural textile assemblies with numerous innovative fabrication techniques, along with various performance enhancement strategies toward highly durable and washable wearable e-textiles. However, challenges related to stability, repeatability, durability, washability, scalability, and other process-induced flaws limit the manufacture and commercialization of customer reliable high-end wearable electronic textiles products (see Figure 11). Therefore, for the e-textiles device to be commercially successful beyond the laboratory, future research should be focused more on the following issues and research gaps to design multipurpose reusable wearable electronic clothing as casual wear at the customer level.

Reliable durability enhancement strategies are to be adopted according to the substrate, nanomaterials, and processing involved in designing the e-textiles device. Different surface modification approaches in the pre-treatment stages involving bio proteins, adhesives, cross-linkers, plasma, and other chemicals should not affect the structural integrity of the textile substrate. Bioproteins as surface modifiers are assumed to be eco-friendly, but other organic cross-linkers and bonding agents may have a higher environmental impact which cannot be unattended. Besides, post-treatments such as encapsulation of the conductive textiles with traditional encapsulants (TPU, PDMS, epoxy resins, etc.) have been proven to be effective in protecting functional properties securely, but in some cases, the lamination layer was found to be vulnerable and washed away. The encapsulation of the e-textiles should not affect the breathability, comfort, flexibility, and other inherent properties of the substrate and should be compatible with human skin.

The internal wiring of the wearable components is very much crucial for the optimum performance of the entire unit but is mostly overlooked and needs more attention. Therefore, a reliable interconnection pattern among different functional units within the wearable system is essential for consistent performance, that is, data acquisition and processing without interruption. Commercial metallic wires (silver, steel, copper, etc.) are mostly explored for e-textile interconnections, but they are stiff, incompatible, and may malfunction under mechanical stresses involved in the wash and wear. The failure or malfunctioning of interconnections can cause short-circuits within the system, which may pose a serious safety threat to the wearer. The flexible electroconductive fibers/yarns can be the best alternative but need to be extensively studied to improve their robustness for interconnections of various patterns. In addition, the seamless integration possibilities of the electronic components into the clothing needs broader investigation toward a robust wearable system.

The scalability of electronic textiles cannot be ignored as it is also directly related to the productivity and cost of the wearable garment. Future research should be more focused on designing e-textiles beyond the laboratory environment at a large scale. Fast and facile manufacturing in combination with traditional textile processing techniques will promote mass production compared to sophisticated laboratory techniques. In addition to the higher endurance properties, the cost-effectiveness of the e-textile products should also be taken into account for potential market expansion. Moreover, inclusive simulation and modeling of current techniques (substrate treatment, nanomaterial incorporation, post-treatment, washing, drying, and product design) are required to achieve greater efficiency and in fact to develop new strategies.

Electronic textile fabrication involves various organic or inorganic chemical treatments (surfactants, nanoparticles, polymers, metals, acids, bases, etc.) in a wet medium, which can release substantial amounts of toxic elements to the environment and even pose a significant threat to the consumer such as skin irritation or carcinogenic disease when in contact with the human body. Superior washability of e-textiles will promote the lower toxic release to the body when encountered with a wet environment (sweating, bleeding, and raining) during wearing. However, the risk of toxic release both to the environment and the human body is so evident that it cannot be ignored, and greater attention is required for a more sustainable and cleaner approach. As the e-textile market continues to grow, a huge burden of countless used wearable electronic textile materials is expected to be added to the current solid waste chain in the coming years. Such waste is significantly more toxic and dangerous than the solid waste generated from regular textile wear; therefore, a sustainable and eco-friendly solid E-waste management is required.

Traditional domestic laundry involves a huge amount of water which may contain toxic chemicals released from e-textiles during washing, and the release of such contaminated water into the environment without further purification may have a catastrophic effect on the aquatic ecosystem. Waterless washing techniques will prevent such a level of pollution by lowering the water footprint, but may also increase the operating cost associated with alternative approaches. The environmental compatibility of dry-cleaning chemicals should be investigated. Each washing technique (wet/dry) may have a distinctive impact on the properties of the substrate; therefore, it is important to understand the washing damages for a particular type of material to be washed following a specific technique. More research should be devoted to the synchronization of different washing techniques with the type, structure, and composition of the e-textile product to be laundered to minimize washing damages.

Imparting high-end functionality such as self-cleaning properties toward e-textiles will significantly reduce their washing needs, as they are expected to repel and decompose dirt through photocatalytic action. The superhydrophobic surface achieved via the coating of different chemicals is also capable of repelling or removing dirt, dust, and other impurities by rolling water droplets inspired by the lotus effect. Although various chemical compounds have been explored to exert such functionalities on e-textile components, they should be biocompatible and not affect the comfort and aesthetic properties. The environmentally friendly fluorine-free chemical reagents or polymers can replace traditional toxic hydrophobic coatings but require more research for a better understanding of the cleaning mechanism and efficiency. The self-healing property, i.e., automatic repairing of different stimuli-induced damages, will substantially improve the robustness of the e-textiles and make them more viable for practical application. Therefore, extensive research is required on the development of self-healable polymers and their performance under different hostile events throughout the life span of the e-textile components.

Despite the efforts of different international organizations to standardize e-textiles’ washing protocols, researchers should focus on mitigating the underlying mismatch in materials, structure, fabrication, and product design to validate and adopt forthcoming standards widely. The stability and repeatability of the device performance cannot be ignored, which needs to be given the same priority as washability and requires standardized documentation. It is very possible that an e-textile component is claimed to be washable but may exhibit an unstable, irregular, and unreproducible performance profile for a prolonged duration. Flexible, lightweight e-textile batteries in fibrous shape are promising and are expected to replace traditional rigid and heavy power sources embedded in wearables, but the efficient power generation and management, mobility, and endurance of such flexible devices require more research attention. Moreover, future work should also address the accuracy and reliability of the data measurement of the durable wearable electronic textile system in the context of practical applications.

## Figures and Tables

**Table 1:** Summary of different yarn-shaped durable wearable electronic textiles.

Substrate	Nano Materials	Fabrication	Initial Output	Durability		Application	Ref.
				Stability	Washability		
Pu/PAN core- sheath yarn	GO/CNT ink	Dip Coating	Conductivity, 14.8 S m^−1^	~100,000 operation cycles, 99.3% capacitance retention	5 cycles, no significant deterioration of capacitance	Pressure sensor, motion sensing	[161]
Cotton/Lycra yarn	CNT	Dip coating	Resistance, 2.39 kΩ cm^−1^	~Cyclic stretching-releasing for 2000 s, high stability	10 cycles, slight increase of resistance (ΔR/R_0_ ~ 1.6)	Strain sensing, thermal heating	[165]
Pu/PET braided yarn	CNT	Dip Coating	Conductivity, 0.12 kΩ cm^−1^	~1000 stretch-release cycles, no obvious change in resistance	5 cycles, slight increase (ΔR/R_0_ ~ 10%) of resistance	Wearable strain sensor	[166]
PET yarn	Cu	Electroless deposition	Resistance, 0.34 Ω cm^−1^	~1000 tapping cycles, no change of voltage output	20 cycles, negligible change (<0.6 Ω cm^−1^) of yarn resistance	Respiratory Monitoring	[167]
SS/terylene yarn	SS filament	Spinning	Output voltage, 28 V	~100,000 loading-unloading cycles, excellent stability	40 cycles, no change of output voltage	Physiological signal monitoring	[168]
Nylon yarn	Silver	Nano coating	Resistance, 53 Ω m^−1^	-	50 cycles, notable resistance change (108%)	Biomedical textile computing	[169]
Lyocell yarn	PPy	Polymerization	Conductivity, 21.6 Ω Sq^−1^	~2000 cyclic operations, 90% capacitance retention	20 cycles, minor variations in electrical response	Wearable electronics	[170]
Cotton yarn	RGO	Dip Coating	Conductance (2.60 ± 0.1 μS)	~1000 bending cycles, slight variation (2.42%) in conductance	5 cycles, minimal (2.96% variation) conductance change	Gas sensing	[171]
CNT yarn	CNT, PEI, FeCl_3_	CVD, Doping	Conductivity, 3695 S cm^−1^	~5000 bending cycles, retained 90% PCE	10 cycles, slight change of PCE	Solar cell	[172]
Silk yarn	PEDOT:PSS, EG	Roll to roll dyeing	Conductivity, 70 S cm^−1^	~1000 bending cycles, stable resistance profile	15 cycles, slight change after 1st wash than resistance kept constant	Wearable keyboard	[173]
Cotton yarn	RGO	Dip Coating	Resistance, 42.7 kΩ cm^−1^	~1000 bending and compression cycles, stable resistance variance	10 cycles, resistance increased initially then kept constant	Temperature sensor	[174]
Silver-plated nylon yarn	CNTs, TPU	Electrospinning	Sensitivity, 84.5 N^−1^	~5000 pressure (5 N) cycles, stable current signal obtained	2.5 h of washing, constant order of magnitude (only 1.4% variation)	Pressure sensor	[175]

Abbreviation: Pu—Polyurethane, PAN—Polyacrylonitrile, GO—Graphene oxide, RGO—Reduced graphene oxide, CNT—Carbon nanotube, PET—Polyethylene terephthalate, SS—Stainless steel, PPy—Polypyrrole, PEI—Polyethyleneimine, CVD—Chemical vapor deposition, PCE—Power conversion efficiency, PEDOT: PSS—Poly (3,4-ethylenedioxythiophene) polystyrene sulfonate, EG—Ethylene glycol, TPU—Thermoplastic polyurethane.

**Table 2:** Summary of different fabric shaped durable wearable electronic textiles.

Substrate	Nano Materials	Fabrication	Initial Output	Durability		Application	Ref.
				Stability	Washability		
Nylon Fabric	SWCNT, MoO_3_	Spray Coating	Resistance, 8.55 MΩ	~10,000 stretching-releasing cycles, outstanding stability	10 cycles, no change of relative resistance	Supercapacitor	[188]
PET Fabric	Ag ink	Inkjet Printing	Conductivity, 0.9 ± 0.02 Ω·sq^−1^	~10,000 bending cycles, no significant change of resistance	15 cycles, resistance increased by 2 times of initial resistance	Conductive Textiles	[178]
Nanofiber membrane	Poly(Ionic Liquid)	Electrospinning	Resistance, 3 × 10^6^ Ω·sq^−1^	~300 loading/unloading cycles, no capacitance degradation	10 cycles, consistent performance	Pressure sensor	[189]
Woven Fabric	SS core yarn	Weaving	Power density, 9.9 μWm^−2^	~4200 pressing cycles, with no degradation of current output	4 h washing, constant electrical output (voltage)	Triboelectric sensing	[190]
Cotton Fabric	PEDOT:PSS	Screen Printing	Resistance, 22.70 kΩ	-	50 cycles, maintained similar ECG wave pattern	ECG electrode	[191]
Nylon Fabric	CNT	Screen Printing	Conductivity, 0.2 kΩ·sq^−1^	~2000 bending cycles, no obvious change in resistance	10 h of water immersion, negligible changes in resistance	Self-powered gesture sensor	[192]
Cotton Fabric	RGO/ SWCNT	Dip Coating	Gauge factor, 5.4	~100,000 bending (11.6% strain) cycles, excellent stability	10 cycles, no change of surface resistance	Motion sensor	[193]
Textile Fabric	Gold nanowire	Dip Coating	Resistance, 12.4 MΩ	30,000 Sec of loading-unloading cycles, constant output signal	48 h of washing, a slight increase (7.3%) of resistance	Health monitoring	[194]
Cotton Fabric	RGO	Dip Coating	Sheet resistance, 0.9 kΩ·sq^−1^	~400 bending cycles, Stable resistance change	10 cycles, slight increase of resistance (0.9 to 1.2 kΩ/sq)	Strain sensor	[195]
Cotton Fabric	Ag nanowire	Dip and Dry	Power output, 1.25 Wm^−2^	~3000 cyclic bendings, no change of voltage output (Voc)	15 cycles, output voltages of the electrode preserved well	Nanogenerator	[196]
Wool Fabric	RGO	Pad Dyeing	Sheet resistance, 12.3 kΩ·sq^−1^	~500 stretch-release cycles, steady change of relative resistance	10 cycles, moderate increase of resistance (14 to 20.5 kΩ/sq)	Strain sensing	[197]
Cotton Fabric	PAH, Cu, F-POSS/POTS	Deposition	Sheet resistance, 0.33 Ω·sq^−1^	~5000 bending cycles, slight change of resistance (0.52 ± 0.18 Ω·sq^−1^)	100 cycles, Conductivity maintained well (0.32 Ω·sq^−^^1^)	Self-cleaning, E-textiles	[198]

Abbreviation: SWCNT—Single-walled carbon nanotube, MoO_3_—Molybdenum trioxide, SS—Stainless steel, PEDOT: PSS—Poly (3,4-ethylenedioxythiophene) Polystyrene sulfonate, RGO—Reduced graphene oxide, PAH—Poly (allylamine hydrochloride), F-POSS; Fluorinated-decyl polyhedral oligomeric silsesquioxane, POTS—1H,1H,2H,2H-perfluorooctyltriethoxysilane.

**Table 3:** Durability of e-textiles achieved by different surface modification techniques.

Substrate	Modification	Nano Materials	Fabrication	Initial Output	Durability		Ref.
					Stability	Washability	
Cotton Fabric	PDA	Ag NW	Dip Coating	Resistance, 7.12 Ω/cm	~2000 bending cycles, constant resistance change	10 cycles, insignificant change of resistance	[223]
Nylon 6 Yarn	BSA	RGO	Electrostatic assembly	Conductivity (>1000 S/m)	~400 bending cycles, negligible variations in conductivity	9 cycles, no significant change of conductivity	[224]
PET Substrate	Plasma Treatment	AgNW, GO	Blade Coating	Conductivity (>20 Ω/Sq.)	~700 bending/stretching cycles, slight change in ΔR (%)	6 cycles, no change of sheet resistance	[225]
PP nonwoven	Plasma Treatment	PEDOT:TOS	Immersion Coating	Conductivity, 2.19 S/cm	~300 bending cycles, 20% loss of electrical resistance	3 h of washing, conductivity lost and got stable after 1.5 h	[226]
Polyester Fabric	PVA	MXene	Dip Coating	Resistance, 930 Ω	~1000 loading/unloading cycles, no changes in resistance	30 min washing, good washability	[227]
Cotton Fabric	Ink with PVA binder	CB	Dip Coating	Resistance, 25–28 kΩ/Sq.	~1000 bending cycles, durable and reliable performance	12 cycles, resistance increased initially but was stable	[228]
Cotton Yarn	β-lactoglobulin	RGO	Dip Coating	Conductance, 0.91 ± 0.32 μS	~1000 bending cycles, slight changes (~1.47% in SD)	5 cycles, no dramatic change, (∼0.063 μA in SD)	[229]
Cotton Fabric	GMA grafting and APA	PANI	In-situ polymerization	Resistance, 2× 10^9^ Ω/Sq.	Reversible conductivity switching (5 cycles) behavior	40 cycles, with almost no change of conductivity	[230]
Nylon 6 Fabric	PA/APTES	Cu	Electroless deposition	Resistance, 0.0056 Ω/Sq.	~1000 bending cycles, stable performance (R/R_0_ ~ 2.1)	50 cycles, slight increase in sheet resistance	[231]
Cotton Fabric	MPTS	Silver	Electroless deposition	Resistance, 0.33 Ω/Sq.	-	200 cycles, slight increase of resistance (to 2.49 Ω/Sq.)	[232]
PET Fabric	GA	GO	Laser Scribing	Capacitance (756 µFcm^−2^)	~1000 operation cycles, 98.3% capacitance retention	Good wash fastness properties	[233]
Cotton Yarn	Polyelectrolyte brushes	Cu	Electroless deposition	Conductivity, 1 S/cm	~30 stretch/release cycles, unchanged conductivity	5 cycles, no degradation of conductivity	[234]

Abbreviation: PDA—Polydopamine, BSA—Bovine serum albumin, PVA—Polyvinyl alcohol, GMA—Glycidyl methacrylate, APA—4 Aminophenethylamine, PA/APTES—Phatic acid/Aminopropyltriethoxysilane, MPTS—3 Mercaptopropytrimethoxysilane, GA—glutaraldehyde, CB—Carbon black, PEDOT:TOS—Poly(3,4-ethylene dioxythiophene):p-toluenesulfonic acid, PANI—Polyaniline, R/GO—Reduced/Graphene oxide, NW—Nanowire4.2. Encapsulation.

**Table 4:** Durability enhancement of e-textiles through different encapsulation techniques.

Substrate	Nano materials	Encapsulant	Initial Output	Durability		Ref.
				Stability	Washability	
Cotton Fabric	RGO	PE773	Conductivity, 11.9Ω/Sq.	15,000 operation cycles, 98% capacitance retention	10 cycles, 3.5 times increase in resistance	[241]
PU Yarn	AgNW	Eco-flex	Sensitivity, 0.136 kPa^−1^	5000 loading/unloading cycles (0.05 kPa pressure), stable output signals	10 cycles, 14% reduction of capacitance	[242]
Cotton Fabric	MXene	PDMS	Conductivity, 126 S/m	500 loading/unloading cycles (20% strain), sensing signal hardly changed	5 h of ultrasonic washing, maintained hydrophobicity (~147°) well	[243]
PET Fabric	GO ink	HDI	Planar resistance, 861 Ω/sq	500 stretching-releasing cycles (10% strain), stable negative response	120 min laundry, insignificant loss of conductivity	[244]
PET Fiber	PEDOT	PMMA	Electric resistance, 600 Ω cm^−1^	1000 stretching-releasing cycles, gauge factor became relatively stable	-	[245]
Cotton Fabric	Ag, Cu	Silicon	Resistance, 6 Ω/in.	500 bending cycles, constant electrical resistance profile	8 laboratory washing, resistance drastically changed for unencapsulated substrate	[246]
Textile Substrate	Polymer Solar cell	Acrylic adhesive	Current density, 14.85 mA cm^−2^	1000 repeated bending cycles, insignificant changes in output	20 cycles, retained 98% of initial efficiency	[247]
Polymer substrate	Organic photovoltaics	Parylene	PCE, 7.9%	Cyclic compression (43%), 99% PCE retained	120 min water immersion (5.4% decrease in efficiency)	[248]
Cotton Fabric	Ag NW	NOA63	Sheet resistance, 12 Ω/Sq.	500 bending cycles, no change in luminance	50 washing cycles, electroluminescence remained almost unchanged	[249]
TPU Nonwoven	GO, CNC	Hf-SiO2	Guage Factor, 2.36 × 10^4^	1000 tensile cycles at 10% strain, good stability (no apparent fluctuation)	20 cycles, encapsulated substrate kept unchanged, R/R_0_ ~ 15 for bare substrate	[250]
Woven Fabric	PEDOT:PSS, RGO	EG, DMSO	Sheet resistance, 10–15 Ω	10,000 bending cycles, no degradation in sheet resistance	25 washing cycles, sheet resistance increased from 20 Ω to 90 Ω	[251]
Cotton Fabric	PPy, MXene	HDTMS	Water contact angle, 158°	Long-term stability (>1000 bending cycles)	5 h ultrasonication, WCA decreased to 144° but remained hydrophobic	[252]
Spacer Fabric	SWCNT, Ag	DM-SIP-2500	Sensitivity, 4.2 × 10^−2^ kPa^−1^	20,000 loading/unloading cycles, uniform capacitive changes (<7%)	45 laundry cycles, slight changes (<8.05%) in capacitance	[253]

Abbreviation: PE773—Commercial encapsulant, PDMS—Polydimethylsiloxane, HDI—Hexamethylene diisocyanate, PMMA—Polymethyl methacrylate, NOA63—Norland optical adhesive, Hf-SiO2—Hydrophobic fumed silica, EG—Ethylene glycol, DMSO—Dimethyl sulfoxide, HDTMS—Hexadecyltrimethoxysilane, CNC—Cellulose nanocrystal, PCE—Power conversion efficiency, DM-SIP-2500—Commercial encapsulation paste.

**Table 5:** Different washing strategies in absence of standardized durability assessment protocols for e-textiles.

Textiles	Washing Tech.	Standard	Parameters							Ref.
			Time	Temp.	Deterg.	Load	Rotation	Wash	Drying	
Cotton fiber/CNT	Container wash	AATCC 61-2006	45 min	40 °C	200 mL	10 SS ball	40 ± 2 rpm	8 Cyc.	Hang dry	[147]
Cotton yarn/CB	Beaker Wash	-	10 min	25 °C	1 wt%	-	Magnetic Stirr	16 Cyc.	Air Dry	[153]
Cellulose yarn/PEDOT:PSS-EG	Domestic Laundry M/C	Wool Program	-	30 °C	Yes	-	800 rpm	10 Cyc.	-	[154]
Nylon/MWCNT;AgNW	Beaker Wash	-	10 min	60 °C	-	-	300 rpm Stirr	5 Cyc.	-	[159]
SS-PET yarn	M/C Wash	-	20 min	30 °C	-	-	-	8 Cyc.	Hanging	[160]
PAN fiber/rGO;CNT	Hand Wash	-	5 min	25 °C	4 g/L	-	-	5 Cyc.	Air Dry	[161]
PDVF/CNT yarn	Beaker wash	ISO 6330 A7	10 min	30 °C	No	-	400 rpm	10 Cyc	Air dry	[163]
PET yarn/Cu	M/C Wash	AATCC 135, Hand laundry program	40 min	20 °C	Yes	1.8 kg	119 strokes/min, 430 rpm	20 Cyc.	Air hang dry	[167]
SS-Terylene yarn	Commercial M/C wash	AATCC 135	5 min	25 °C	Yes	1.8 kg	119 strokes/min, 430 rpm	40 Cyc.	Air hang dry	[168]
PET fabric/Silver ink	Canister wash	AATCC 61	45 min	49 °C	0.24 gm	50 SS ball	-	15 Cyc.	50 °C, 15 min	[187]
PET Fabric/RGO, CU_2_O	Domestic wash	GB/T 12490	30 min	40 °C	NO	10 SS ball	-	40 Cyc.	Room temp.	[209]
PI fabric/TPU-MWCNT	Beaker wash	ISO 105-C03	60 min	60 °C	0.37 wt%	-	Ultrasonication	20 Cyc	60 °C, oven	[219]
Cotton Fabric/PANI	Dry wash	AATCC 86-2005	30 min	30 °C	200 mL TTE		Intense Stirring	40 Cyc.	Air Dry	[230]
Nylon Fabric/Cu	M/C wash	GB/T 5454–1997	30 min	40 °C	5 g/L	-	-	50 Cyc	-	[231]
Silk yarn/PEDOT:PSS	M/C Wash	Hand wash prog.	50 min	30 °C	20 mL	-	900 rpm	4 Cyc.	Line Dry	[254]
PET braided yarn/CNT	Beaker wash	-	30 min	-	-	-	Ultrasonication	5 Cyc.	Vacuum dry	[166]
PI fabric/PEDOT:PSS	Household M/C	ISO 6330	35 min	40 °C	30 mL	2.5 kg	600 rpm	50 Cyc.	-	[255]
Solar cell/Textiles	Hand wash	AATCC M5	-	-	Yes	-	-	25 Cyc.	Line dry	[256]
Cu and Ni fabric/PDMS	Hand scrubbing	AATCC-138 2005	-	50 °C	0.3 mL	-	-	7 Cyc.	Oven dry	[257]

Abbreviation: Temp.—Temperature, Deterg.—Detergent, CYC.—Cycles, M/C—Machine, SS—Stainless Steel, CB—Carbon black, NW—Nanowire, PANI—Polyaniline.

**Table 6:** Summary of different existing and forthcoming e-textile standards.

Org.	Test Method	Details	Status	Ref.
ISO	FDIS 24584	Test method for sheet resistance of conductive textiles using non-contact type	Under development	[274]
AATCC	TM210	Test method for electrical resistance before and after various exposure conditions (laundering, dry cleaning, water, perspiration, acids and alkalis, ultraviolet (UV) radiation, and/or microbes)	Published in 2019	[275]
	EP13	Evaluation procedure for electrical resistance of electronically integrated textiles	Published in 2018 (Revised 2021)	[276]
ASTM	WK61480	Method for durability of smart garment textile electrodes after laundering	Under Development	[277]
	D8248-20	Standard terminology for smart textiles	Published in 2020	[278]
IEC	63203-101-1	Terminology used in wearable electronic devices and technologies	Published in 2021	[279]
	63203-201-3	Determination of electrical resistance of conductive textiles under simulated microclimate (air layer containing humidity and temperature between skin and clothing)	Published in 2021	[280]
	63203-204-1	Test method for assessing washing durability of leisurewear and sportswear e-textile systems	Under development, expected release in mid-2023	[281]
	TR 63203-250-1	Snap fastener connectors between e-textiles and detachable electronic devices	Published in 2021	[282]
	63203-406-1	Test method for measuring surface temperature of wrist-worn wearable electronic devices while in contact with human skin	Published in 2021	[283]
IPC	8921	Requirements for woven and knitted electronic textiles (e-textiles) integrated with conductive fibers, conductive yarns, and/or wires	Published in 2019	[284]
	8921A	Requirements for woven, knitted, and braided electronic textiles (e-textiles) integrated with conductive yarns and/or wires	Not published yet	
	JPCA-8911	Requirements for conductive yarns for e-textiles applications	Under development	
	8952	Design standard for printed electronics on coated or treated textiles and e-textiles	Under development, Expected release by the end of 2022	
	8971	Requirements for electrical testing of printed electronics e-textiles	Under development, Expected release in mid-2022	
	8981	Quality and reliability for e-textiles wearables	Under development, Expected release in early 2023	
	WP-024	IPC White Paper on Reliability and Washability of Smart Textile Structures—Readiness for the Market	Published in 2018	[285]
	WP-025	IPC White Paper on A Framework for the Engineering and Design of E-Textiles	Published in 2019	[286]

Abbreviation: Org.—Organization, ISO—International Organization for Standardization, AATCC—American Association of Textile Chemists and Colorists, ASTM—American Society for Testing and Materials, IEC—International Electrotechnical Commission, IPC—Institute of Printed Circuits.

## Data Availability

Not applicable.
